# Trans-Ancestral Fine-Mapping and Epigenetic Annotation as Tools to Delineate Functionally Relevant Risk Alleles at *IKZF1* and *IKZF3* in Systemic Lupus Erythematosus

**DOI:** 10.3390/ijms21218383

**Published:** 2020-11-09

**Authors:** Timothy J. Vyse, Deborah S. Cunninghame Graham

**Affiliations:** Department of Medical and Molecular Genetics, King’s College London, London SE1 9RT, UK; timothy.vyse@kcl.ac.uk

**Keywords:** trans-ancestral fine-mapping, Systemic Lupus Erythematosus, epigenetics, functional annotation

## Abstract

**Background:** Prioritizing tag-SNPs carried on extended risk haplotypes at susceptibility loci for common disease is a challenge. **Methods:** We utilized trans-ancestral exclusion mapping to reduce risk haplotypes at *IKZF1* and *IKZF3* identified in multiple ancestries from SLE GWAS and ImmunoChip datasets. We characterized functional annotation data across each risk haplotype from publicly available datasets including ENCODE, RoadMap Consortium, PC Hi-C data from 3D genome browser, NESDR NTR conditional eQTL database, GeneCards Genehancers and TF (transcription factor) binding sites from Haploregv4. **Results:** We refined the 60 kb associated haplotype upstream of *IKZF1* to just 12 tag-SNPs tagging a 47.7 kb core risk haplotype. There was preferential enrichment of DNAse I hypersensitivity and H3K27ac modification across the 3′ end of the risk haplotype, with four tag-SNPs sharing allele-specific TF binding sites with promoter variants, which are eQTLs for *IKZF1* in whole blood. At *IKZF3*, we refined a core risk haplotype of 101 kb (27 tag-SNPs) from an initial extended haplotype of 194 kb (282 tag-SNPs), which had widespread DNAse I hypersensitivity, H3K27ac modification and multiple allele-specific TF binding sites. Dimerization of Fox family TFs bound at the 3′ and promoter of *IKZF3* may stabilize chromatin looping across the locus. **Conclusions:** We combined trans-ancestral exclusion mapping and epigenetic annotation to identify variants at both *IKZF1* and *IKZF3* with the highest likelihood of biological relevance. The approach will be of strong interest to other complex trait geneticists seeking to attribute biological relevance to risk alleles on extended risk haplotypes in their disease of interest.

## 1. Introduction

***Systemic Lupus Erythematosus (SLE)*** is a complex autoimmune disease of unknown etiology. However, genome-wide association analysis of cohorts has proven to be a successful means of identifying novel susceptibility loci for lupus [1,2,3,4,5,6,7,8,9,10,11]. The 84 autosomal genetic risk factors identified in the largest of these Genome-wide association studies (GWAS) studies, in a Euro-Canadian cohort [12]) implicate many different gene families from diverse biochemical pathways. Dysregulation of these molecular pathways could have serious consequences for the function of multiple immune cell types. The Ikaros family of Kruppel zinc finger transcription factors is one such gene family. The importance of this gene family in SLE pathogenesis is evidenced by the associations (*P_meta_* < 5 × 10^−8^) for three family members: *IKZF1* (Ikaros) (rs2366293-C, rs4917014-T), *IKZF3* (Aiolos) (rs2941509-T) and *IKZF2* (Helios) (rs6435760-C) [12].

The Ikaros transcription factors are important regulatory proteins in hematopoiesis and lymphocyte function and as such make good functional candidates for lupus. Excluding Pegasus (*IKZF5*) the other four member of the Ikaros transcription factor gene family co-evolved in pairs: *IKZF1* and *IKZF3* from a common ancestor IKFL1 and *IKZF2* (Helios) and *IKZF4* (Eos) from IKFL2 [13]. However, all four proteins have subsequently developed functional differences and expression profiles. The focus of this manuscript the trans-ancestral fine mapping and epigenetic characterization of the two IKFL1-derived IKZF transcription factors, namely *IKZF3* and *IKZF1*. There is strong evidence to support both *IKZF1* and *IKZF3* as strong candidates for SLE. Expression of *IKZF3* is largely restricted to T and B cells and the Aiolos knockout mouse, which spontaneously develops a lupus-like phenotype, is characterized by the chronic activation of B cells with increased levels of autoantibodies and glomerulonephritis [14]. *IKZF1* has a wider expression pattern in blood cell types, being involved in hematopoietic stem cell development [15] and in lymphoid development, as evidenced by the lack of T, B, NK and dendritic cells in a mouse model which lacks *Ikzf1* DNA-binding exons 3–5 [16]. Myeloid cell types are unaffected.

Both IKZF3 and IKZF1 Have also Been Reported to be Risk Factors for Other Autoimmune Diseases. At IKZF1, although associations have been reported for multiple autoimmune diseases, there is no common consensus risk variant between studies for SLE and: Crohn’s Disease (rs1456896) [17]; Irritable Bowel Disease (rs1456896) [18]; Ulcerative Colitis (rs1456896) [18], Multiple Sclerosis (rs201847125) [19], Type I Diabetes (rs10272724) [20]. The associated in variant in SLE (rs4917014) has limited linkage disequilibrium (LD) (r^2^ = 0.25) with any of the variants for the other autoimmune diseases listed and is present at a higher minor allele frequency (MAF) than the other AID variants in Europeans.

The association at the *IKZF3* locus in European SLE is different from that seen in the other autoimmune diseases, where the association is driven by a high frequency (MAF > 40% risk allele): Crohn’s Disease (rs2872507, rs12946510) [17,18]; Rheumatoid Arthritis (rs2872507) [21]; Primary Biliary Cirrhosis (rs8067378) [22]; Ulcerative Colitis (rs12946510, rs2872507) [18,23]; Multiple Sclerosis (rs12946510) [19]; Inflammatory Bowel Disease (rs12946510) [18]; Childhood Asthma (multiple variants) [24] or T1D (rs12453507) [25]. None of these variants is in LD with the SLE variant (r^2^ < 0.03) and the non-SLE variants show strong LD (r^2^ > 0.80) with each other.

In the literature, there is no convincing data to support a role for rs4917014 as a conclusive *cis*-eQTL for *IKZF1*. There is a single report, comparing IKZF1 protein expression in different types of B cells from SLE cases (n = 10) and healthy controls (n = 10). There was a marginal increase in the MFI detection for IKZF1+ CD27^+^IgD^−^ switched memory (SwM) B cells, CD27^+^IgD^+^ double-positive non-switched memory (NSM) B cells and CD27^−^IgD^−^ DN B cells in SLE patients compared with healthy controls. In the same dataset there was less MFI detected for CD27^−^IgD^+^ mature naive B cells in the patients compared with the healthy controls [26]. Therefore, acknowledging that this existing protein expression data uses both limited cell types and activity states and that the results were not correlated with genetic risk factors, we looked for evidence of other mechanisms whereby risk alleles at *IKZF1* may influence *IKZF1* levels.

The risk alleles for both *IKZF1* and *IKZF3* lie on extended haplotypes, which makes it challenging to define causal variants for functional studies. In this paper a combined approach to identify risk alleles with an increased likelihood for biologic function. Firstly, we annotate tag-SNPs on the risk haplotypes at both loci using publicly available epigenetic and regulatory datasets, from Roadmap [27], ENCODE [28], PC-Hi-C [29] and Haploreg v4 [30]. Those alleles carried on risk haplotypes which possess or are co-localized with, a greater level of epigenetic modification are more likely to have functional significance. The second part of our strategy capitalizes on the differential severity and prevalence of SLE between ancestries. We use a trans-ancestral fine-mapping method to define shared variants on population-specific haplotypes, which increases the weight in prioritization for functional characterization. Therefore, using a “two-pronged attack” exploiting both epigenetic annotation and trans-ancestral fine mapping we seek to narrow down the core regions of association at *IKZF1* and *IKZF3* and define sets of candidate causal variants at each locus.

## 2. Results

### 2.1. Defining the Risk Haplotype at IKZF1 in SLE

**Figure A1:**
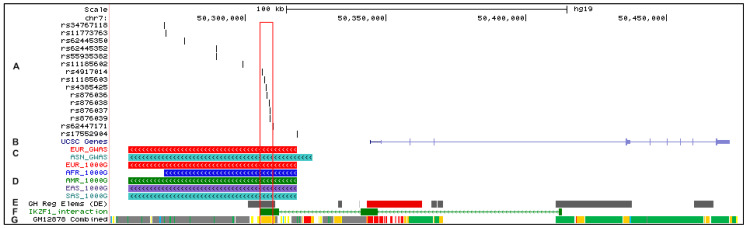
Trans-ancestral fine-mapping of the *IKZF1* risk haplotype. The diagram illustrates the power of trans-ancestral fine mapping at *IKZF1*. **Panel A:** Illustrates the associated SNPs in the 47 kb core risk haplotype following trans-ancestral alignment of the *IKZF1* haplotypes. Each variant is in strong LD (r^2^ > 0.75) with rs4917014 (*P_meta_* < 5 × 10^−8^). **Panel B:** Position of the core risk haplotype in relation to the genomic architecture across *IKZF1*. **Panels C and D:** Datasets used for defining the core risk haplotype. **Panel C:** Location of 60 kb full “risk” haplotype in healthy controls from the European GWAS (EUR_GWAS) with that from two Chinese GWAS (ASN_GWAS)—comprising variants in strong LD (r^2^ > 0.75) with rs4917014. **Panel D:** Alignment of the “risk” haplotypes in healthy individuals from the five super-populations of the 1000G project comprising variants in strong LD (r^2^ > 0.75) with rs4917014: ***EUR_1000G*** (shown in red); ***AFR_1000G*** (show in blue); ***AMR_1000G*** (shown in green); ***SAS_1000G*** (shown in turquoise) and ***EAS_1000G*** (shown in purple). The dashed box delineates the 47 kb core share haplotype bounded by rs34767118 and rs876039 (chr7:50271064-50308811). **Panel E:** GeneHancer regulatory elements at *IKZF1* from GeneCards—from left to right: GH07J050261 (chr7:50300992-50310765); GH07J050293 (chr7:50333047-50334464); GH07J050301 (chr7:50340632-50340761); GH07J050303 (chr7:50343395-50362927); GH07J050326 (chr7:50366368-50368325); GH07J050329 (chr7:50368690-50370631); GH07J050341 (chr7:50410631-50437890) and GH07J050392 (chr7:50459865-50466852). The Promoter/TSS interval is designated as a red box and the enhancer intervals as grey boxes. **Panel F:** Interaction regions at *IKZF1* from Left to Right: ***Enhancer*** (Enh) (chr7:50305428-50311993); ***Transcriptional Start Site/Promoter*** (TSS) (chr7:50341186-50347256) and ***intron 3*** (I3) (chr7:50411807-50412756) [29]. **Panel G:** Combined Genome Segmentation data from ENCODE in EBV-LCLs. All seven variants lying within the risk haplotype (bounded by a red box, lie within a region predicted to be an enhancer (orange).

The strongest risk allele at *IKZF1* (rs4917014-T) from our European SLE GWAS [12] is located 38.5 kb upstream of the TSS for *IKZF1* (*P_meta_* < 5 × 10^−8^). The variant lies within the proximal end of the risk haplotype in the control samples from this GWAS (Figure A1A–C). This 60 kb risk haplotype (EUR_GWAS) (Figure A1D), which carries a total of 186 variants (using boundary cut-off of r^2^ > 0.75 with rs4917014) is bounded by rs1870027 and rs17552904 (chr7:50258234-50318308, hg19).

The association was replicated in a meta-analysis with two Chinese (ASN) GWAS [7,31,32]. In these Chinese datasets, rs4917014 is located on an overlapping, albeit slightly longer risk haplotype ASN_GWAS, comprising 198 variants over 65 kb, bounded by rs4598207 and rs6964608 (chr7:50258479-50324037, hg19) (Figure A1C). There are no other associations outside these risk haplotypes in either the European or Chinese populations.

The trans-ancestral SLE ImmunoChip study [33] provided minimal additional information, because the gene-centric genotyping platform used for the study had sparse coverage of the *IKZF1* risk haplotype. Only five of the variants on the risk haplotypes from the European/Chinese GWAS studies were included on the chip. However, the dataset revealed that the MAF of those five risk alleles were more similar in samples of European and Asian origin to those of African origin. There was association for all five variants in African Americans and European samples (Table A1). We cannot explore the association in African samples in more detail because there is currently no published SLE GWAS in samples of African origin.

### 2.2. Refining the IKZF1 Risk Haplotype Using the 1000 Genomes Super-Populations

**Figure 1:**
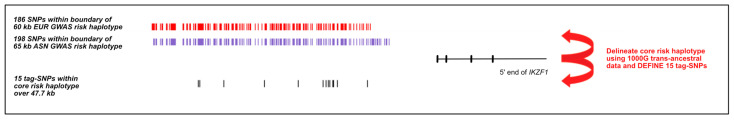
Trans-ancestral mapping to define a core set of *IKZF1* risk alleles. The figure shows the location of the 186 SNPs defined within the boundary of the 60 kb *IKZF1* risk haplotype and the 198 SNPs within the 65 kb Chinese (ASN) risk haplotype. Alignment of the 1000G haplotypes carrying alleles in LD (r^2^ > 0.75) with rs4917014 (as shown in Figure A1) was used to refine the risk haplotype to 15 variants in tight LD (r^2^ > 0.75) with rs4917014 over a distance of 47.7 kb upstream of the *IKZF1* transcriptional start site.

We narrowed down the risk haplotype with a trans-ancestral mapping approach, using healthy individuals taken from the five superpopulations from the 1000G super-population data: AFR—African; AMR—Admixed American; EAS—East Asian; EUR—European and SAS—South Asian. The refined region around rs4917014 shared across ancestries, using an LD cut-off of r^2^ > 0.75 with rs4917014, comprised 15 SNPs across only 47.7 kb, bounded by rs34767118 and rs876039 (chr7:50271064-50308811) (Figure 1). This region is most likely to harbor alleles of functional significance at *IKZF1*.

### 2.3. Functional Annotation of IKZF1 Risk Alleles

Given the limited cell types used for the published protein expression data in SLE samples [26] and the fact that the authors did not select cells based on specific risk alleles at *IKZF1*, we employed several strategies to investigate the mechanisms by which risk alleles may impact *IKZF1* expression levels. We used publicly available epigenetic data in a diverse set of immune cell types to search for enrichment of epigenetic signals which overlapped the risk alleles within the 47.7 kb *IKZF1* risk haplotype and therefore more likely to have functional significance.

#### 2.3.1. Determination of Chromatin Status

Alignment of the risk alleles upstream of *IKZF1* revealed that only the seven SNPs on the risk haplotype lie within a predicted enhancer (orange) using the Combined Genome Segmentation data from ENCODE in LCLs (Figure A1G). The remaining five variants were located within areas of heterochromatin (grey) or low activity (green). Taken together, these data suggest that the seven variants within the predicted enhancer region are more likely to be functionally active.

#### 2.3.2. Chromatin Looping with Risk Alleles

**Figure 2:**
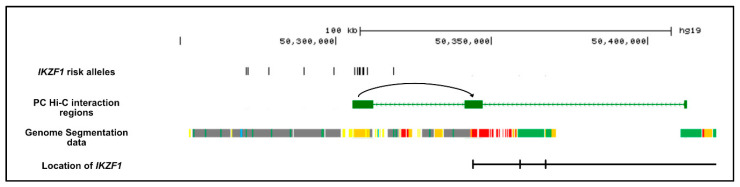
Chromatin Status of *IKZF1* Interaction Regions. The figure shows several aligned tracks across *IKZF1* (hg19). The 15 risk alleles are aligned with the three interaction regions at *IKZF1*, reading from Left to Right: Upstream Enhancer region; proximal promoter (TSS) and intron 3 (I3). There is chromatin looping between the Enhancer region and the TSS region but not intron 3. The Genome Segmentation data was extracted from ENCODE (EBV-LCL), using a merged consensus of the segmentations from ChromHMM and Segway algorithms. The seven states correspond to: ***Predicted promoter including TSS*** (bright red), ***Predicted promoter flanking region*** (light red), ***Predicted enhancer*** (orange), ***Predicted weak enhancer or open chromatin cis regulatory element*** (yellow), ***CTCF enriched element*** (blue), ***Predicted transcribed region*** (Dark Green), ***Predicted Repressed or Low Activity region*** (grey).

**Figure A2:**
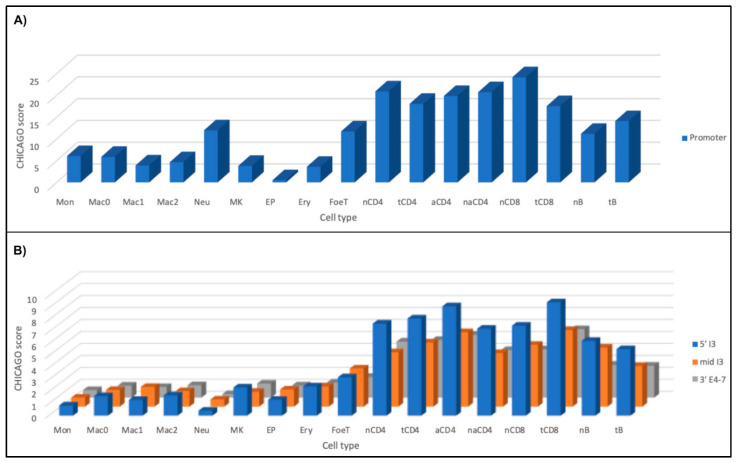
Chromatin looping at *IKZF1* and *IKZF3* in immune cell types. The figure shows the chromatin looping events at (**A**) *IKZF1* and (**B**) *IKZF3* in multiple immune cell types [29]. A CHICAGO score (soft-thresholded -log weighted *p*-values) of >5 represents a significance interaction between two intervals. At *IKZF1*, there was only one chromatin looping event between the ***Promoter (TSS)*** (chr7:50341186-50347256) and the ***Enhancer*** (chr7:50305428-50311993). At *IKZF3*, there are three interaction regions between the bi-directional promoter ***IKZF3-ZPBP2*** (chr17:38018444-38027003) and the coding region of the gene (**5′ I3**) chr17:37965773-37976506; (**mid I3**) chr17:37958027-37963133 and (**3′ E4-7**) chr17:37932293-37957717. The immune cell types analyzed are: ***Monocytes*** (Mon); ***Macrophages M0*** (Mac0); ***Macrophages M1*** (Mac1); ***Macrophages M2*** (Mac2); ***Neutrophils*** (Neu); ***Megakaryocytes*** (MK); ***Endothelial precursors*** (EP); ***Erythroblasts*** (Ery); ***Fetal thymus*** (FoeT); ***Naïve CD4+ T cells*** (nCD4); ***Total CD4+ T cells*** (tCD4); ***Activated total CD4+ T cells*** (aCD4); ***Non-activated total CD4+ T cells*** (naCD4); ***Naïve CD8+ T cells*** (nCD8); ***Total CD8+ T cells*** (tCD8); ***Naïve B cells*** (nB) and ***Total B cells*** (tB).

The *IKZF1* promoter is the hub of chromatin looping events at the locus. Analysis of Promoter Capture Hi-C data showed three interaction regions at *IKZF1 (*Figure 2 and Figure A1F) [29]. These data revealed that the proximal promoter (chr7:50341186-50347256) (TSS) interacts with the 3′ end of the enhancer region (chr7:50305428-50311993) (Enh) in multiple immune cell types (Figure A2A). The Enh region contains a set of seven risk alleles. A second interaction between the TSS and a shorter sequence in intron 3 (chr7:50411807-50412756) (I3) did not involve the Enh region (data not shown). There was cell-type specificity in the Enh-TSS looping activities (Figure A2A), with the strongest interaction (CHICAGO score > 11) seen in neutrophils, T and B lymphocytes. Each of the cell types which exhibited strong interaction also demonstrated higher than median *IKZF1* expression for the human cells/tissues assessed by the GeneAtlas U133A microarray (BIOGPS) [34].

**Figure A3:**
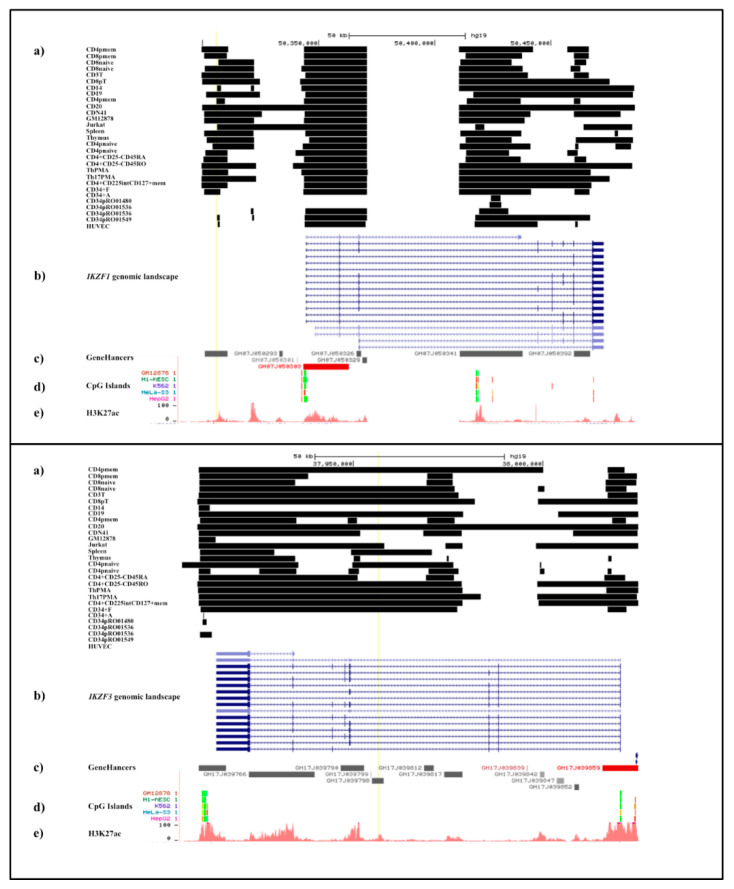
Genomic Landscape of the SuperEnhancers at *IKZF1* and *IKZF3.* The figure illustrates the genomic architecture around the SuperEnhancers at *IKZF1* (chr7:50,289,782-50,486,079) (**top panel**) and *IKZF3* (chr17:37,904,434-38,025,200) (hg19) (**lower panel**). For each locus: (**a**) shows the position of individual enhancer regions was extracted from (Hnisz et al. 2013) [40] for immune cell types and illustrated by black boxes in the following cell types: **CD4pmem**—CD4 primary Memory T cells; **CD8mem**—CD8 memory T cells; **CD8naive**—CD8 naïve T cells; **CD8naive**—CD8 naïve T cells; **CD3T**—CD3 T cells; **CD8pT**—CD8 primary T cells; **CD14**—CD14 cells; **CD19**—CD19 cells; **CD4pmem**—CD4 primary memory T cells; **CD20**—CD20 cells, CD56 cells; **CND41**—CND41 cells; **GM12878**—GM12878; **Jurkat**—Jurkat T cells; **Spleen**—Spleen; **Thymus**—Thymus; **CD4pnaive**—CD4 naïve primary T cells; **CD4pnaive**—CD4 naïve primary T cells; **CD4+CD25-CD45RA**—CD4+ CD25- CD45RA Naïve T cells; **CD4+CD25-CD45RO**—CD4+ CD25- CD45RO T cells, **ThPMA**—CD4+ CD25- Il17- PMA stimulated Th cells; **Th17PMA**—CD4+ CD25- Il17+ PMA stimulated Th17; **CD4+CD225intCD127+mem**—CD4+ CD225int CD127+ memory T cells; **CD34+F**—CD34+ fetal cells; **CD34+A**—CD34+ adult cells; **CD34pRO01480**—CD34 primary RO01480 cells; **CD34pRO01536**—CD34 primary RO01536 cells; **CD34pRO01549**—CD34 primary RO01549 cells; **HUVEC**—HUVEC. (**b**) The transcript isoforms of *IKZF1* and *IKZF3*; (**c**) the GeneHancer regions; (**d**) The location of the CpG islands is illustrated using the CpG track from the UCSC genome browser in several vertebrate cell lines (PMID: 3656447) and (**e**) the H3K27Ac Mark (Often Found Near Active Regulatory Elements) from ENCODE in GM12878 cells.

We also found that the 47.7 kb risk haplotype overlaps with a 9.7 kb GeneHancer region (GH07J050261) designated by the GeneHancer database [35,36]. GH07J050261 contains seven of the *IKZF1* risk alleles (Figure A1E) and there is evidence of chromatin looping events between GH07J050261 and a second GeneHancer interval in the promoter (GH07J050303). The core risk haplotype lies within a previously identified SuperEnhancer region stretching into and across the *IKZF1* coding region for multiple immune cell types (Figure A3).

#### 2.3.3. Cell-Type Specificity in DNAse Sensitivity in the IKZF1 Enhancer Region

**Figure 3:**
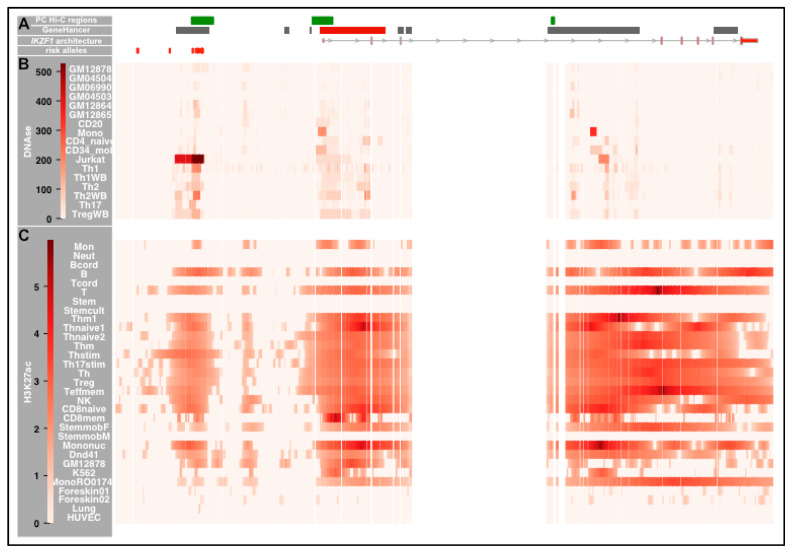
Genomic and Epigenetic Landscape across *IKZF1.* The figure shows the genomic landscape around *IKZF1*. The data is split into three horizontal panels (**A**–**C**). The genomic location of each element is presented in Table A2. **Panel A: *The top row*** PC Hi-C interaction regions from left to right designated: **Enhancer** (Enh); **Transcriptional Start Site/Promoter** (TSS) and **intron 3** (I3). ***The second row*** illustrates the GeneHancer regulatory regions (grey boxes and promoter/TSS regions (red boxes) from GeneCards—from left to right: GH07J050261; GH07J050293; GH07J050301; GH07J050303; GH07J050326; GH07J050329; GH07J050341 and GH07J050392. ***The third row*** illustrates the genomic architecture of the major *IKZF1* transcript. ***The fourth row*** shows the location of the risk alleles at *IKZF1*, which are in strong LD (r^2^ > 0.75) with the GWAS risk variant, rs4917104: rs34767118, rs11773763, rs62445350, rs55935382, rs11185602, rs4917014, rs11185603, rs4385425, rs876036, rs876038, rs876037 and rs876039). **Panel B:** heatmaps delineating the Signal Values of the DNAse Hotspots, calculated by the Sato et al. 2004 method. These data were taken from Digital DNAseI data from ENCODE/Washington for immune cells: ***GM12878*** (EBV-LCL); ***GM04504*** (EBV-LCL); ***GM06990*** (EBV-LCL); ***GM04503*** (EBV-LCL); ***GM12864*** (EBV-LCL); ***GM12865*** (EBV-LCL); ***CD20*** (CD20+ B cells); ***Mono*** (CD14+ Monocytes); ***CD4*** (naïve CD4+ T cells from whole blood); ***CD34+*** (Mobilized CD34+ cells); ***Jurkat*** (Jurkat T cell line); ***Th1*** (purified Th1 cells); ***Th1WB*** (Th1 cells from whole blood); ***Th2*** (purified Th1 cells); ***Th2WB*** (Th1 cells from whole blood); ***Th17*** (T helper cells expressing IL-17) and ***Treg*** (Regulatory T cells). **Panel C:** heatmaps illustrating the enrichment of the H3K27ac enhancer mark (using the consolidated imputed epigenetic data in RoadMap), calculated by the IntervalStats tool in the Colocstats web browser. The blood cell types from RoadMap are: ***Mon*** (E029—Primary monocytes from peripheral blood); ***Neut*** (E030—Primary neutrophils from peripheral blood); ***Bcord*** (E031—Primary B cells from cord blood); ***B*** (E032—Primary B cells from peripheral blood); ***Tcord*** (E033 and E034—Primary T cells from cord blood); ***T*** (E034—Primary T cells from peripheral blood); ***Stem*** (E035—Primary hematopoietic stem cells); ***Stemcult*** (E036—Primary hematopoietic stem cells short term culture); ***Thm1*** (E037—Primary T helper memory cells from peripheral blood); ***Thnaive1*** (E038—Primary T helper naive cells from peripheral blood); ***Thnaive2*** (E039—Primary T helper naive cells from peripheral blood); ***Thm2*** (E040—Primary T helper memory cells from peripheral blood); ***Thstim*** (E041—Primary T helper cells PMA-I stimulated); ***Th17stim*** (E042—Primary T helper 17 cells PMA-I stimulated); ***Th*** (E043—Primary T helper cells from peripheral blood); ***Treg*** (E044—Primary T regulatory cells from peripheral blood); ***Teffmem*** (E045—Prim. T cells effector/memory enriched from periph. Blood); ***NK*** (E046—Primary Natural Killer cells from peripheral blood); ***CD8naive*** (E047—Primary T CD8+ naïve cells from peripheral blood); ***CD8mem*** (E048—Primary T CD8+ memory cells from peripheral blood); ***StemmobF*** (E050—Primary hematopoietic stem cells G-CSF-mobilized Female); ***StemmobM*** (E051—Primary hematopoietic stem cells G-CSF-mobilized Male); ***Mononuc*** (E062—Primary mononuclear cells from peripheral blood); ***Dnd41*** (E115—Dnd41 TCell Leukemia Cell Line); ***GM12878*** (E116—GM12878 Lymphoblastoid Cell Line); ***K562*** (E123—K562 Leukemia Cell Line) and ***MonoRO01746*** (E124—Monocytes-CD14+ RO01746 Primary Cells). The non-blood cells from RoadMap are ***Forekin01*** (E055—Foreskin Fibroblast Primary Cells), ***Forekin02*** (E055—Foreskin Fibroblast Primary Cells), ***Lung*** (E128—NHLF Lung Fibroblast Primary Cells) and ***HUVEC*** (E122—HUVEC Umbilical Vein Endothelial Primary Cells).

**Figure A4:**
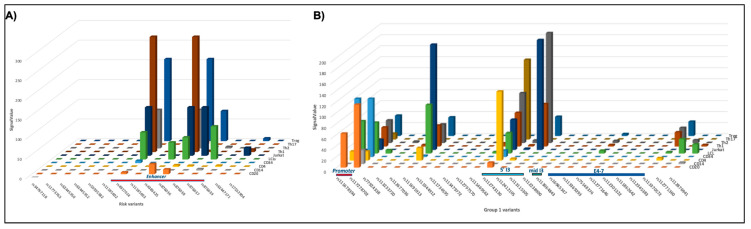
DNAse Hotspots across risk variants at *IKZF1* and *IKZF3* in immune cells. The figure displays the SignalValues of the DNA Hotspots for (**A**) the core risk variants at *IKZF1* and (**B**) Group I variants at *IKZF3,* in the following immune cell types taken from ENCODE: **CD20**—CD20+ B cells (RO01778); **CD14**—Monocytes CD14+ RO01746; **CD4**—CD4+ T cells_Naive_Wb11970640, CD4+_ T cells Naive_Wb78495824; **CD34**—CD34+ Mobilized; **LCL**—EBV-LCL (GM12865, GM12864, GM06990, GM04504, GM04503); **Jurkat**—Jurkat cells; **Th1**—Th1, Th1_Wb54553204, Th1_Wb33676984; **Th2**—Th2, Th2_Wb54553204, Th2_Wb33676984; **Th17**—Th17 cells; **T reg**—Treg_ Wb83319432, Treg_Wb78495824. The location of the interaction regions from PC Hi-C is illustrated above the variants for *IKZF1:* ***Enhancer*** (Enh) (chr7:50305428-50311993) and *IKZF3*: Promoter (chr17:38018444-38027003) with the three interaction regions across the coding region chr17:37965773-37976506 (**5′ I3**); chr17:37958027-37963133 (**mid I3**) and chr17:37932293-37957717 (**3′ E4-7**).

Figure 3 demonstrates preferential enrichment of DNAse I across *IKZF1* in T cells. The PC Hi-C enhancer region exhibits the most convincing DNAse I hotspots (SignalValue > 5), with the strongest signals being in Th1 cells and regulatory T cells at rs4917014 and rs876036 (Figure A4A).

#### 2.3.4. Discovery of Allele-Specific Transcription Factor Binding Sites

We characterized the transcription factors which are predicted to show allele-specific differences in binding affinity (from Haploreg v4.1) to each of the 12 risk alleles defined by GWAS. Ten of these polymorphism are predicted to exhibit allele-specific binding of one or more TFs (Table A3). Five of the risk alleles within the PC Hi-C Enh region exhibit strong allele-specific binding affinity (>3 fold predicted change) for TFs which also bind to variants in the *IKZF1* PC Hi-C TSS/promoter interaction region or the GeneHancer promoter region (Table 1). These five risk variants, through shared binding events have the greatest potential for genetic control of *IKZF1* gene expression through chromatin looping events, leading to dimerization of the shared TF and increased regulatory activity on gene expression.

**Figure 4:**
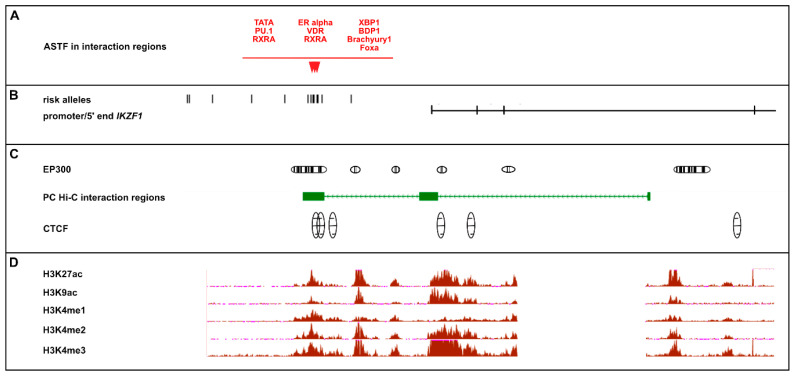
Epigenetic Annotation of Risk Alleles at *IKZF1.* The figure is a diagrammatic representation summarizing the functional annotation across *IKZF1*. All of the data in Panels A-D was prepared in a single alignment against hg19 (chr7:50,279,064-50,481,386). **Panel A**: The transcription factors which are predicted to exhibit significant (LOD < 3) allele-specific binding (ASTF) to *IKZF1* risk alleles within the PC-Hi-C interaction regions, taken from Table 1. **Panel B**: Genomic architecture of *IKZF1* and the location of the 15 upstream risk alleles. **Panel C**: Clusters of statistically significant enrichment (score range 200–1000) ChIP-Seq peaks for EP300 and CTCF (Transcription Factor ChIP-seq Uniform Peaks from ENCODE/Analysis) in GM12878 EBV-LCLs, aligned with the PC-Hi-C interaction intervals across IKZF3. **Panel D**: ChIP-Seq signal wiggle density graphs for chromatin marks from ENCODE/BROAD in GM12878 EBV-LCL cells for-H3K27ac (active enhancer region), H3K9ac (active regulatory elements/promoters), H3K4me1 (found in gene body of CpG genes with higher expression), H3K4me2 (found in gene body of CpG genes with higher expression) and H3K4me3 (associated with promoter/TSS). The vertical viewing range for each of these epigenetic tracks is set to viewing maximum at 50, to allow comparison of signal between each epigenetic modification.

Figure 4 summarizes the epigenetic landscape across *IKZF1.* The TFs predicted to show allele-specific binding (ASTF) lie within one of the CTCF regions within the upstream associated region and at one of the multiple EP300 binding sites across the locus. Both of these elements are characteristic of enhancer regions. There is also evidence of several epigenetic modifications across the region which commonly reside in active enhancers (H3K27ac), active regulatory elements/promoters (H3K9ac); promoter/TSS (H3K4me3) or are located in the gene body of CpG genes with higher expression (H3K4me1 and H3K4me2).

#### 2.3.5. Identification of cis-eQTLs at *IKZF1*

None of the SLE risk alleles in the PC Hi-C Enh or TSS/Promoter regions are themselves *cis*-eQTLs for *IKZF1* expression in whole blood from the GTEx2015_v6 data or from the NESDR NTR conditional eQTL database [37,38].

However, four of the ten risk variants predicted to exhibit allele-specific TF binding share the same TFs with other polymorphism in the promoter GH07J050293 interaction region, which are also *cis*-eQTLs for *IKZF1* in whole blood in either the GTEx2015_v6(*) or the NESDA NTR conditional eQTL(#) databases (Table 1). These six promoter eQTLs are: rs11765436/rs7802443-RXRA-***rs11185603***; rs9886239-PU.1-***rs11185603***; rs11761922/rs7781977-BDP1-***rs876038***; rs10269380-Brachyury-***rs876038*** and rs7777365-FOXA-***rs876039***. It will be important to establish whether the TFs involved form a “bridge” to support the chromatin looping between the enhancer and promoter regions and whether there is a potential contribution of SLE risk alleles to control gene expression at *IKZF1*.

### 2.4. Extended IKZF3 Haplotype across Multiple Genes in European SLE GWAS Study

**Figure A5:**
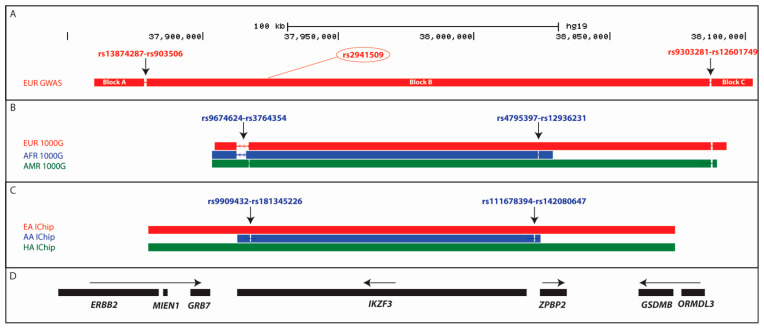
Trans-ancestral Fine-Mapping of *IKZF3.* All of the data in **Panels A**–**D** are from a single alignment from the various studies analyzed in this manuscript. (**A**) shows the haplotype block structure across the *IKZF3* locus constructed using 15,991 healthy individuals from a European SLE GWAS [12]. ***Block B*** represents the 194 kb region covering the ~3% risk haplotype, carrying the *IKZF3* risk variant from the GWAS (rs2941509) (chr17:37879762-38074046). ***Blocks A and C*** are the adjacent haplotype blocks in which there are no associated variants. The SNPs delineating the break-down in LD between the haplotype blocks A and B and between B and C are shown (rs13874287-rs903506 and rs9303281-rs12601749 respectively). There is no LD between any of the SNPs in block A and any of the associated variants in block B (r^2^ < 0.02) and between any of the associated SNPs in block B compared to any variants in block C (r^2^ < 0.03). (**B**) Alignment of haplotypes across *IKZF3* in the European (EUR—shown in red), African (AFR—shown in blue) and Amerindian (AMR—shown in green) super-populations from the 1000 Genomes project. The 107 kb LD block shared by all three super-populations which carries rs2941509 is bounded by two LD breakpoints (rs9909432-rs181345226 and rs111678394-rs142080647) (chr17:37916823-38023745). (**C**) The haplotype structure across *IKZF3* is shown in the healthy controls from the SLE ImmunoChip dataset, comprising 11,516 European-American (EA), 2452 African-American (AA) and 2016 Hispanic-American (HA) samples. The 101 kb shared risk haplotype carrying rs2914509 is bounded by two LD breakpoints (rs9909432-rs181345226 and rs111678394-rs142080647) (chr17:37920146-38021117). (**D**) This panel shows the location of the protein coding genes across the locus, with arrows designating the direction of transcription.

In our European GWAS [12] we identified a single associated haplotype at the *IKZF3* locus which stretches from intron 19 of *ERBB2* (rs903506), across *IKZF3*, *ZPBP2*, *GSDMB* and *ORMDL3* into the upstream region of *ORMDL3* (rs9303281) (Figure A5A), a distance 194 kb (chr17:37879762-38074046). This European *IKZF3* risk haplotype (EUR-*IKZF3* haplotype) present at a frequency of 3% in Europeans, is tagged by the minor risk alleles of 282 variants with each of the five genes within the haplotype boundary containing multiple risk alleles. The peak association from conditional analyses is in the 3′ UTR of *IKZF3* (rs2941509). However, the tight LD across the locus in Europeans means that it is not possible to discriminate between any of the 282 tag SNPs as possessing functional significance.

### 2.5. Fine-Mapping the IKZF3 Risk Haplotype Using the 1000 Genomes Super-Populations

In an attempt to narrow down the region of the European risk haplotype to define the segment most likely to harbor alleles of functional significance, we adopted a trans-ancestral approach, which utilized the five 1000G super-population datasets, to discover the minimal risk haplotype shared between ancestries.

The frequency of the European risk haplotype in the EUR-GWAS (3%) and EUR 1000G samples (2.9%) is ~6-fold less in the African AFR 1000G samples (12.5%), whereas in AMR individuals the frequency was marginally below (2.3%) that seen in EUR samples. In both Asian super-populations, the EUR-*IKZF3* haplotype was present at <0.1%, so we did not include the two Asian super-populations in further trans-ancestral analyses.

The alignment of the haplotype blocks from AFR, EUR and AMR 1000G samples allowed us to identify a common shared haplotype block containing the rs2941509 risk variant, of 107 kb (Figure A5B). In all three datasets, the 3′ of this refined haplotype is at the 3′ end of *IKZF3*, between the immediate 3′ flanking region (within an *IKZF1* ChIP-binding site from ENCODE in EBV-LCLs) (rs9674624) and the 3′ UTR (rs3764354). The 5′ boundary of the risk haplotype was defined using the AFR 1000G samples because in both EUR and AMR samples the 5′ LD break is in the same place, upstream of *ORMDL3* (rs112191651-rs4795405). However, in the AFR samples, the haplotype block is shorter, with the 5′ boundary lying within an *IKZF1* binding site in the *IKZF3*-*ZPBP2* bi-directional promoter (rs4795397-rs12936231). Taken together, these results show that the AFR samples are a key discriminator in narrowing down the common shared haplotype. Using the 1000G data we have successfully reduced the length of the core *IKZF3* risk haplotype by over 44% from 194 kb (EUR GWAS) to 107 kb (AFR 1000G)(chr17:37916823-38023745). We have also reduced the number of tag SNPs from 282 (EUR GWAS) to 152 (AFR 1000G) (Figure A5B).

Using genotypes from 2452 AA healthy control samples on the ImmunoChip we further reduced the length of the risk haplotype block, at both the 5′ and 3′ ends, by a total of 6 kb compared to the same block in the AFR 1000 Genomes dataset (Figure A5B). In a similar manner to our results in the AFR 1000G samples, the haplotype carrying the European risk alleles (EUR-IKZF3 haplotype) in the AA (African-American) ImmunoChip cohort was present at a higher frequency (~12%) than in European samples. However, in the HA (Hispanic-American) (n_controls_ = 2016) ImmunoChip cohort, the haplotype carrying the European risk alleles was at a reduced frequency (2.5%) compared to the European GWAS haplotype (Figure A5B), albeit it the same length, so would not add any further information in fine-mapping the European signal.

In summary, the LD break-points in both the AA ImmunoChip and AFR 1000G datasets allow us to massively reduce, by >47%, the *IKZF3* risk haplotype first identified in the Euro-Canadian SLE GWAS, leading to a risk haplotype covering 101 kb (chr17:37920146-38021117), restricted to the coding region for *IKZF3* and carrying only 140 European tag-SNPs.

### 2.6. Trans-Ancestral Exclusion Mapping of IKZF3 using the SLE ImmunoChip Data

We replicated the association signal at *IKZF3* in a EA (European-American) SLE ImmunoChip cohort (n_cases_ = 6748, n_controls_ = 11,516), with a total of 93 tag-SNPs in LD with rs2941509 (OR_rs2941509_ = 1.27, CI 1.14–1.41) showing highly significant association (Table A4).

We used trans-ancestral exclusion mapping as a method of narrowing down the EUR-*IKZF3* risk haplotype to variants with greater potential for biological significance, by excluding sets of variants based on the strength of association and MAF in two ancestries. Our analyses split the associated variants into two groups with 27 of the 93 tagging variants (Group 1) showing association with SLE (OR > 1.27) in the AA ImmunoChip cohort (n_cases_ = 2970, n_controls_ = 2452). The remaining 66 variants (Group 2) were not associated (OR < 1.14) with lupus in AA samples. None of the Group 1 or 2 variants were associated with other autoimmune diseases (from the GWAS Catalogue).

Furthermore, the Group 1 risk alleles (3.6% in EA samples) were much rarer in the AA ImmunoChip cohort (MAF < 0.1%). Conversely, for the Group 2 variants, the risk alleles from the EA study present at a higher MAF in the AA cohort (MAF >12%) compared with the EA samples. However, the increased frequency of the Group 2 variants did not lead to increased association in the AA population. Added to this, meta-analysis of the EA and AFR ImmunoChip datasets revealed that the OR of Group 2 SNPs was not increased by either a fixed effects (OR) or by random effects (OR(R)) model and we found high heterogeneity between the two ancestries (I > 50) (Table A4). These results led to the exclusion of 66 variants on the risk haplotype which included the lead SNP identified in our original GWAS study (rs2941509) [12]. Therefore, we focused our further functional annotation on the 27 Group 1 SNPs because they showed association in both populations and were more likely to harbor alleles of functional significance for lupus.

**Figure 5:**
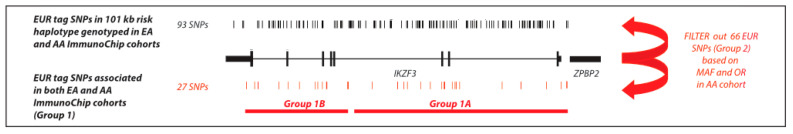
Trans-ancestral exclusion mapping to refine risk alleles at *IKZF3.* Location of the 93 European tag-SNPs carried on the 101 kb core risk haplotype across *IKZF3* coded on the antisense strand, shared between healthy EA (European American) and AA (African American) individuals from the SLE ImmunoChip study. Trans-ancestral exclusion mapping led to the removal of 66 variants (Group 2) which had MAF > 12% but which were not associated (*p* > 0.01) in the AA samples. The remaining 27 variants (Group 1) showed stronger association in the AA samples, despite having MAF < 0.1%. This group of variants, were split into Group 1A (variants located in promoter-I3 regulatory region of the gene) and Group 1B (variants in the I3-E7 region covering the six Zinc Fingers). Group 1A variants were more strongly associated (OR > 1.5) than the Group 1B variants (OR > 1.27) in the AA cohort.

We employed a subsequent round of trans-ancestral exclusion mapping to split the remaining 27 group 1 variants into two sets, based on the degree of association in the AA cohort (Table A4, Figure 5). The 17 variants in Group 1A, which extend across the regulatory region of the gene (between the promoter region and I3), exhibited a stronger association (OR > 1.5) in the AA cohort compared to that seen in the EA population (OR > 1.27). This is despite the meta-analysis of Class 1A variants only providing marginal improvement in association, because of the low MAF in the AA cohort for these SNPs (Table 2). Conversely, the nine SNPs in Group 1B, which lie within the coding region including all six Zinc Fingers (I3-E7), showed similar strength of association in the AA and EA samples, despite the radically reduced MAF for variants in the AA cohort. We will include both Group 1A and 1B variants in our functional annotation of *IKZF3* but have greater confidence that the variants in Group 1A will have a better predictive ability of biological significance than those in Group 1B.

### 2.7. Functional Annotation of Risk Alleles at IKZF3

#### 2.7.1. Analysis of Expression Levels

As with *IKZF1*, none of the *IKZF3* risk alleles are *cis*-eQTLs for *IKZF3* in whole blood [37,38]. At *IKZF3*, this may reflect the lack of power in cis-eQTL analysis given the low MAF of the risk alleles (MAF = 0.03). However, at the protein level there is a significant increase of in the MFI detection of IKZF3 positive CD27^+^IgD^−^ switched memory (SwM) B cells and CD27^+^IgD^+^ double-positive non-switched memory (NSM) B cells in 10 SLE cases and 10 healthy controls, with moderate increases in the detection of MFI in CD27^−^IgD^−^ DN B cells and CD27^−^IgD^+^ mature naive B cells (naive) in the patients compared with the healthy controls [26].

Nevertheless, recognizing that the risk alleles at *IKZF3* may exert their function through epigenetic mechanisms rather than direct transcriptional regulation and that this function may be cell-type and/or activation state specific, we looked for epigenetic mechanisms operating across the risk haplotype which may indicate that specific risk alleles may act in this way.

#### 2.7.2. Determination of Chromatin Looping at IKZF3

**Figure 6:**
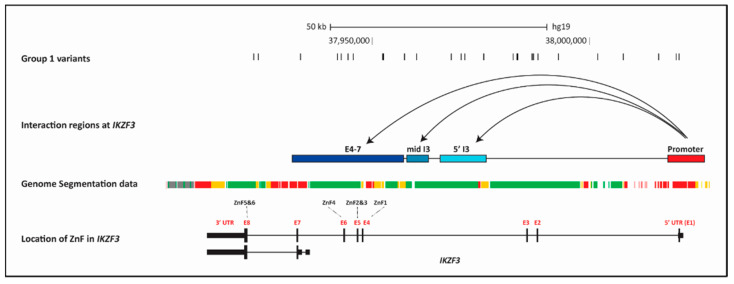
Chromatin Status of *IKZF3* Interaction Regions. The figure shows several aligned tracks across IKZF3 (hg19). The 27 Group 1 variants, aligned with the interaction regions at *IKZF3*: ***IKZF3-ZPBP2*** bi-directional promoter (chr17:38018444-38027003) with the three interaction regions across the coding region chr17:37965773-37976506 (**5′ I3**); chr17:37958027-37963133 (**mid I3**) and chr17:37932293-37957717 (**3′ E4-7**) across *IKZF3*, taken from Pi-HiC data [29]. The strongest interactions (CHICAGO Score > 5.5) were seen in T and B lymphocytes: ***Naïve CD4+ T cells*** (nCD4), ***Total CD4+ T cells*** (tCD4), ***Activated total CD4+ T cells*** (aCD4), ***Non-activated total CD4+ T cells*** (naCD4), ***Naïve CD8+ T cells*** (nCD8), ***Total CD8+ T cells*** (tCD8), ***Naïve B cells*** (nB) and ***Total B cells*** (tB). The Genome Segmentation data was extracted from ENCODE (EBV-LCL), using a merged consensus of the segmentations from ChromHMM and Segway algorithms. The seven states correspond to: ***Predicted promoter including TSS*** (bright red), ***Predicted promoter flanking region*** (light red), ***Predicted enhancer*** (orange), ***Predicted weak enhancer or open chromatin cis regulatory element*** (yellow), ***CTCF enriched element*** (blue), ***Predicted transcribed region*** (Dark Green), ***Predicted Repressed or Low Activity region*** (grey). The genomic architecture of *IKZF3* shows the regions of the gene coding for the Zinc Fingers responsible for DNA binding (***ZnF 1–4***) and dimerization (***ZnF 5–6***). By contrast, there are a total of 12 regulatory elements across *IKZF3* listed in the GeneHancer database (Figure 3, Table A5). However, only one of the GeneHancer elements within *IKZF3* undertakes chromatin looping with the major bi-directional *IKZF3* promoter (GH17J039859). This element is the second promoter (GH17039839), located in intron 1, which contains the ribosomal protein L39 pseudogene 4 (interaction confidence score = 190) (data not shown). (GH17J039859) contains three Group 1 risk alleles but GH17039839 does not contain any risk alleles) (Table A5). Nevertheless, the bi-directional *IKZF3* promoter (GH17J039859) interacts with GeneHancer element upstream of *GSDMB* and *ORMDL3* (GH17J039916) (interaction confidence score = 652). GH17J039916 lies within the original 194 kb EUR associated LD region but not the 101 kb core risk haplotype.

Using the data from the PC Hi-C database, we identified chromatin looping events between the ***IKZF3-ZPBP2*** bi-directional promoter region (chr17:38018444-38027003) and three separate segments within the coding region of the gene: **(5′ I3)** chr17:37965773-37976506; **(mid I3)** chr17:37958027-37963133 in intron 3 and **(3′ E4-7)** (chr17:37932293-37957717 (Figure 3 and Figure 6).

The strongest interactions were between the *IKZF3*-*ZPBP2* promoter and the most 3′ interaction fragment (3′ E4-7) were in naïve CD4+ T cells, total CD4+ T cells, activated total CD4+ T cells, non-activated total CD4+ T cells, naïve CD8+ T cells, total CD8+ T cells, naïve B cells and total B cells (tB) (CHICAGO interaction score > 5.5) (Figure A2B). This 3′ E4-7 interaction region contains the four DNA binding zinc fingers (ZnF 1–4) and the first ~8.4 kb, around the TSS, of a shorter *IKZF3* isoform, implying the promoter-gene interaction may affect the expression of these two functional regions of the locus. Interactions of the promoter with all three coding fragments are greatest in lymphocytes, which reflects the predominant lymphocyte expression pattern of *IKZF3*. However, the 3′ E4-7 interaction region does not contain the two dimerization Zinc Fingers (ZnF 5–6) (Figure 6). The lack of direct interaction between the promoter and dimerization domains means that the risk alleles in the promoter region may only have an indirect interaction with variants in the dimerization domains (in E8) [29,39].

#### 2.7.3. Accessibility of the Chromatin across IKZF3

Extracting the Combined Genome Segmentation data from ENCODE in LCLs, revealed that the entire *IKZF3* risk haplotype is within regions of open chromatin (Figure 6). We also found that the Group 1 variants were preferentially enriched within the three PC Hi-C interaction regions (17 out of 27 SNPs) (Table A4), giving further evidence of potential biological function for these risk alleles. By contrast, although there are 12 GeneHancer regions across *IKZF3*, which contain 17 Group 1 variants, of the two GeneHancer (promoter) regions interacting at *IKZF3* only one of these, the GH17J039859 primary promoter, contained risk alleles (Table A5).

#### 2.7.4. Cell-Type Specificity in DNAse Sensitivity in the IKZF3 Interaction Regions

**Figure 7:**
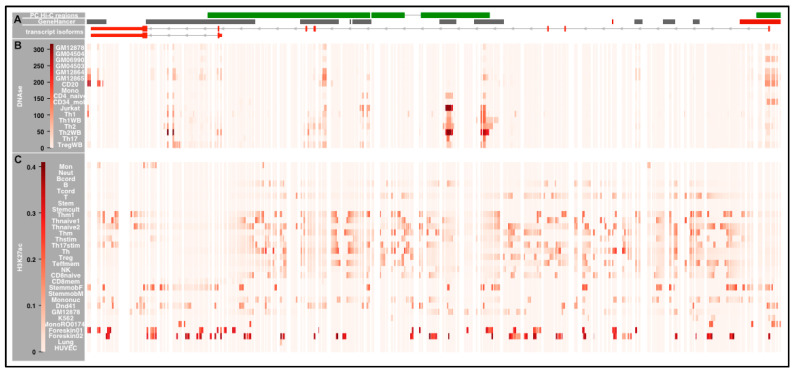
Genomic and Epigenetic Landscape across *IKZF3.* The figure shows the genomic landscape around *IKZF3*. The data is split into three horizontal panels (**A**–**C**). The genomic location of each element is presented in Table A2. **Panel A: *The top row*** PC Hi-C interaction regions from right to left designated: ***IKZF3-ZPBP2*** bi-directional promoter with the three interaction regions across the coding region (**5′ I3**); (**mid I3**) and (**3′ E4-7**). ***The second row*** GeneHancer regulatory elements—from right to right: GH17J039753; GH17J039766; GH17J039790; GH17J039799; GH17J039798; GH17J039812; GH17J039817; GH17J039839; GH17J039842 and GH17J039847. The Promoter/TSS intervals are designated as red boxes and the enhancer intervals as grey boxes. ***The third row*** illustrates the genomic architecture of the full length and short *IKZF3* transcripts. **Panel B:** heatmaps delineating the Signal Values of the DNAse Hotspots, calculated by the Sato et al. 2004 method. These data were taken from Digital DNAseI data from ENCODE/Washington for immune cells: ***GM12878*** (EBV-LCL); ***GM04504*** (EBV-LCL); ***GM06990*** (EBV-LCL); ***GM04503*** (EBV-LCL); ***GM12864*** (EBV-LCL); ***GM12865*** (EBV-LCL); ***CD20*** (CD20+ B cells); ***Mono*** (CD14+ Monocytes); ***CD4*** (naïve CD4+ T cells from whole blood); ***CD34+*** (Mobilized CD34+ cells); ***Jurkat*** (Jurkat T cell line); ***Th1*** (purified Th1 cells); ***Th1WB*** (Th1 cells from whole blood); ***Th2*** (purified Th1 cells); ***Th2WB*** (Th1 cells from whole blood); ***Th17*** (T helper cells expressing IL-17) and ***Treg*** (Regulatory T cells). **Panel C:** heatmaps illustrating the enrichment of the H3K27ac enhancer mark (using the consolidated imputed epigenetic data in RoadMap), calculated by the IntervalStats tool in the Colocstats web browser. The blood cell types from RoadMap are: ***Mon*** (E029—Primary monocytes from peripheral blood); ***Neut*** (E030—Primary neutrophils from peripheral blood); ***Bcord*** (E031—Primary B cells from cord blood); ***B*** (E032—Primary B cells from peripheral blood); ***Tcord*** (E033 and E034—Primary T cells from cord blood); ***T*** (E034—Primary T cells from peripheral blood); ***Stem*** (E035—Primary hematopoietic stem cells); ***Stemcult*** (E036—Primary hematopoietic stem cells short term culture); ***Thm1*** (E037—Primary T helper memory cells from peripheral blood); ***Thnaive1*** (E038—Primary T helper naive cells from peripheral blood); ***Thnaive2*** (E039—Primary T helper naive cells from peripheral blood); ***Thm2*** (E040—Primary T helper memory cells from peripheral blood); ***Thstim*** (E041—Primary T helper cells PMA-I stimulated); ***Th17stim*** (E042—Primary T helper 17 cells PMA-I stimulated); ***Th*** (E043—Primary T helper cells from peripheral blood); ***Treg*** (E044—Primary T regulatory cells from peripheral blood); ***Teffmem*** (E045—Prim. T cells effector/memory enriched from periph. Blood); ***NK*** (E046—Primary Natural Killer cells from peripheral blood); ***CD8naive*** (E047—Primary T CD8+ naïve cells from peripheral blood); ***CD8mem*** (E048—Primary T CD8+ memory cells from peripheral blood); ***StemmobF*** (E050—Primary hematopoietic stem cells G-CSF-mobilized Female); ***StemmobM*** (E051—Primary hematopoietic stem cells G-CSF-mobilized Male); ***Mononuc*** (E062—Primary mononuclear cells from peripheral blood); ***Dnd41*** (E115—Dnd41 TCell Leukemia Cell Line); ***GM12878*** (E116—GM12878 Lymphoblastoid Cell Line); ***K562*** (E123—K562 Leukemia Cell Line) and ***MonoRO01746*** (E124—Monocytes-CD14+ RO01746 Primary Cells). The non-blood cells from RoadMap are ***Forekin01*** (E055—Foreskin Fibroblast Primary Cells), ***Forekin02*** (E055—Foreskin Fibroblast Primary Cells), ***Lung*** (E128—NHLF Lung Fibroblast Primary Cells) and ***HUVEC*** (E122—HUVEC Umbilical Vein Endothelial Primary Cells).

Figure 7 illustrates the enrichment of DNAseI hotspots at the DNA interaction regions across the whole of *IKZF3* from the PC Hi-C or GeneHancer datasets.

The hotspot signal for individual Group 1 risk alleles mirrors the locus-wide signal so that we can see signal enrichment (SignalValue > 2.5) in 14 Group 1 variants spread across the entire risk haplotype (Figure A4B). The most convincing DNAseI hotspots (SignalValue > 5) were seen at Group 1 SNPs predominantly residing within the promoter (*IKZF3*-*ZPBP2*) and the 5′ I3 regions (PC Hi-C experiments). In terms of cell type specificity, the hotspots in B cells are restricted to the promoter region but there is enhanced enrichment of hotspots seen in T cell types within the coding region, including at rs113370572 within the E4-7 interaction fragment. We therefore established that 26 of the Group 1 SNPs were in regions of open chromatin in lymphoblastoid cell lines LCLs (Figure 6) and that there is a degree of cell-type specificity of DNAse1 HS (Figure A4B).

For each allele of the tag-SNPs on the core associated haplotypes for *IKZF1* and *IKZF3*, we extracted the predicted allele-specific differences in binding affinity of transcription factor (taken from the ENCODE TF Binding experiments) from Haploreg v4.1. These differences were calculated as the change in log-odds (LOD) score between the Ref and Alt alleles for each tag-SNP—using Position Weight Matrices (PWM) for any TF binding motifs overlapping a 29 bp region around each risk allele, which reached a stringency (threshold of *p*  <  4^−8^) for either the Ref or Alt allele [30].

#### 2.7.5. Discovery of Allele-Specific Transcription Factor Binding Sites

We extracted the allele-specific differences in TF binding affinity predicted at each of the Group 1 SNPs from the Haploreg database. These results revealed that 18 Group 1 variants exhibited allele-specific differences in binding affinity for one or more of the transcription factors from ENCODE (AS-TF) (Table 2). The table shows the relative strength of this allele-specific binding (using a between cut-off of log-odds >2) for the minor risk (Alt) allele compared with the non-risk (Ref) allele. Ten of these 18 variants lie within one of the four interaction regions described for *IKZF3* from the PC Hi-C data.

We also found that variants within the *IKZF3*-*ZPBP2* bi-directional promoter (chr17:38,020,431-38,024,500) share TF binding sites with the Group 1 risk alleles within the coding region of *IKZF3* (Table 2). Dimerization between these TFs may be a mechanism to stabilize chromatin looping events [40,41] across *IKZF3* and the promoter region.

**Figure 8:**
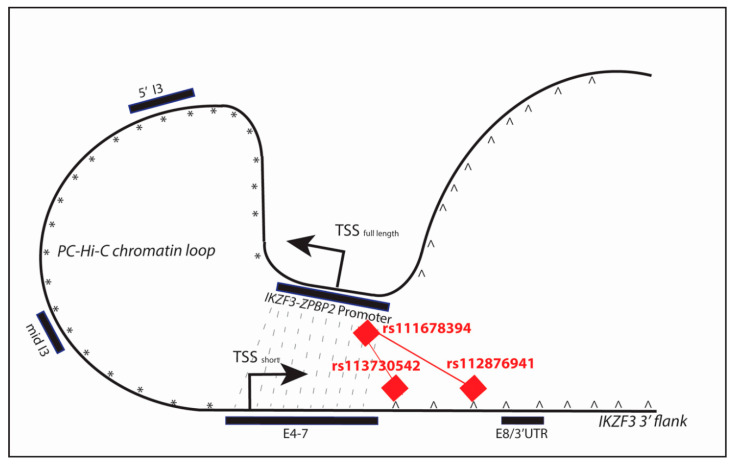
The Potential for Stabilization of Chromatin Looping by TF dimerization at *IKZF3.* The figure illustrates the potential for TF dimerization to stabilize chromatin looping at *IKZF3*. For clarity, we have just shown the interaction between the *IKZF2-ZPBP2* promoter and 3′ E4-7 interaction fragments from PC Hi-C, which brings together the TSS_full length_ (promoter of the full-length isoform) and the TSS_short_ (TSS of the shorter isoform) of *IKZF3* (grey dotted lines). The Fox family members (red diamonds) bind to the risk alleles in the promoter (rs111678394) and dimerize with the Fox TFs binding two risk variants downstream of the 3′ E4-7 fragment: rs113730542 and rs112876941. Since Fox transcription factors act as dimers this potential for Fox dimerization may stabilize the interaction between the *IKZF3-ZPBP2* and 3′ E4-7 fragments.

One example of how TF dimerization may be involved in reinforcing chromatin looping is for the Fox(o) family of transcription factors [42]. Figure 8 illustrates how potential dimerization between members of the Foxo family of TFs, which when bound to three *IKZF3* risk alleles could stabilize chromatin looping across the locus. The *IKZF3-ZPBP2* promoter polymorphism rs111678394 (Foxi1/Foxo_1) can interact with two variants in intron 7: rs113730542 (Fox) (Table A6) and/or rs112876941 (Foxo_2) via Fox family dimerization (Table 2 and Table A7).

#### 2.7.6. IKZF3 Risk Alleles Lie within a SuperEnhancer in B Cells

**Figure 9:**
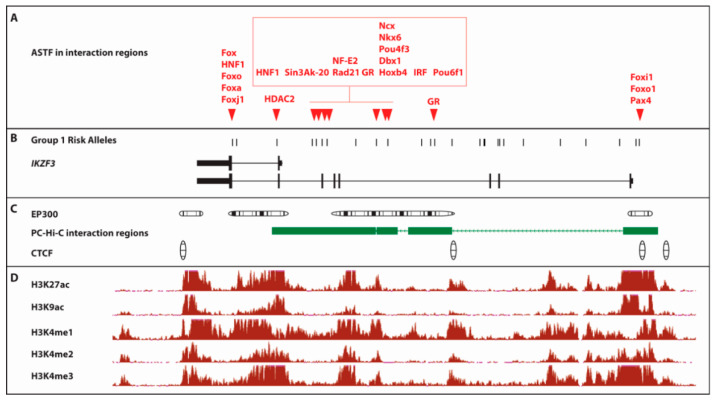
Epigenetic Annotation of Group 1 Risk Alleles at *IKZF3.* The figure is a diagrammatic representation of the functional annotation across *IKZF3*. All of the data in **Panels A**–**D** was prepared in a single alignment against hg19 (chr17:37,892,161-38,035,099). **Panel A**: The transcription factors which are predicted to exhibit significant (LOD < 3) allele-specific binding (ASTF) to group 1 risk alleles within the PC-Hi-C interaction regions, taken from Table 2. **Panel B**: Genomic architecture of IKZF3 and the location of the 26 Group 1 risk alleles (Table 2). **Panel C**: Clusters of statistically significant enrichment (score range 200–1000) ChIP-Seq peaks for EP300 and CTCF (Transcription Factor ChIP-seq Uniform Peaks from ENCODE/Analysis) in GM12878 EBV-LCLs, aligned with the PC-Hi-C interaction intervals across IKZF3. **Panel D**: ChIP-Seq signal wiggle density graphs for chromatin marks from ENCODE/BROAD in GM12878 EBV-LCL cells for—H3K27ac (active enhancer region), H3K9ac (active regulatory elements/promoters), H3K4me1 (found in gene body of CpG genes with higher expression), H3K4me2 (found in gene body of CpG genes with higher expression) and H3K4me3 (associated with promoter/TSS). The vertical viewing range for each of these epigenetic tracks is set to viewing maximum at 50, to allow comparison of signal between each epigenetic modification.

**Figure A6:**
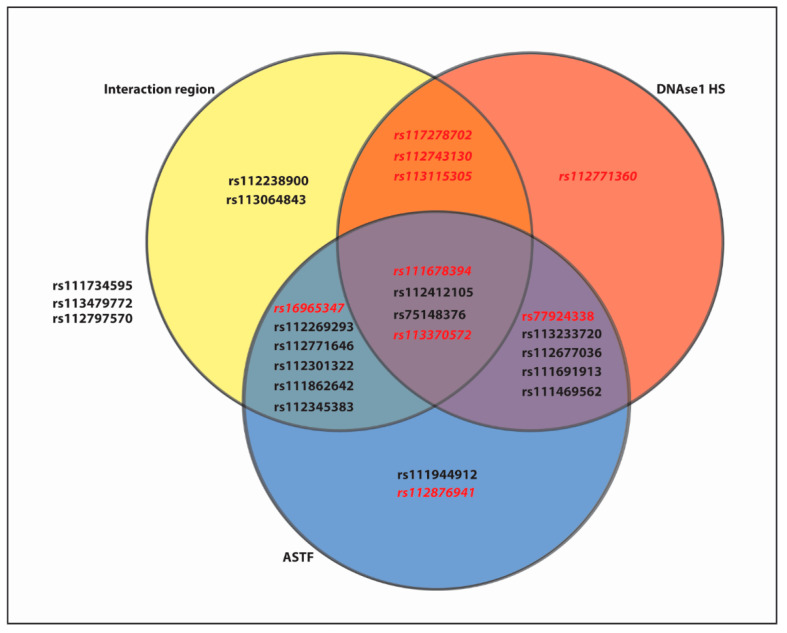
Functional Annotation of Group 1 Variants at *IKZF3.* The figure shows the functional annotation of Group 1 variants. All but three SNPs lie within the annotation categories: **Interaction region**—PC-Hi-C (CHICAGO score > 5); **DNAse1 HS**—DNAse1 hotspot in one or more immune cell types (SignalValue > 2.5) or **AS-TF**—Predicted Allele Specific binding of TF (-log10*P* value > 3). Variants in red, bold text also show enrichment for one or more epigenetic modification (-log10 *p* value > 10).

Figure A6 categorizes the SNP-by-SNP functional annotations across *IKZF3*, revealing that only four variants rs111678394, rs112412105, rs75148376 and rs113370572 lie with a PC Hi-C interaction, a DNAse HS and exhibit a predicted allele-specific TF binding. The variants lie within an interval of just 87.6 kb (chr17: 38,021,116-37,933,467). However, we also know that the entire *IKZF3* region has been identified as a SuperEnhancer in B lymphocytes [43] (Figure A3), which complicates the prioritization of individual variants as having greater functional relevance than others. Some of the additional epigenetic modifications which characterize this SuperEnhancer/core risk haplotype are illustrated in Figure 9. The region is bounded by CTCF binding sites, demonstrating that there is a TAD (topologically associated domain) within *IKZF3* (Figure 9C). We also found multiple EP300 binding sites across the locus, which are also commonly seen in enhancer regions. There are several epigenetic modifications across the entire locus found in EBV-LCLs which characterize: active enhancers (H3K27ac); active regulatory elements/promoters (H3K9ac); promoter/TSS (H3K4me3) or are located in the gene body of CpG genes with higher expression (H3K4me1 and H3K4me2) (Figure 9D).

## 3. Discussion

There is clear evidence from large scale SLE GWAS studies that three members of the Ikaros family of transcription factors (TF) are associated with lupus across multiple ancestries. The Ikaros transcription factors are important regulators of multiple immune cell types but in each case, the risk alleles tag an extended risk haplotype, so the identity of the causal risk alleles is unknown. Identifying these causal risk alleles will be an important step forward in understanding how genetics may alter the function of *IKZF1* and *IKZF3* in SLE.

Since three members of the same family show evidence of association for the same disease, it provides a convincing argument that these TFs play an important role in disease pathogenesis and indeed builds the case for a comprehensive analysis of the association signals in order to define the causal risk alleles at each locus. We therefore used a multi-omic strategy to build up a picture of the genetic, epigenetic and functional annotation across the associated loci, to pin-point the risk alleles which are likely to make the strongest contribution to the genetic-dysregulation of *IKZF1* and *IKZF3*. At each locus we identified a set of risk alleles across multiple ancestries which are located within regions of open chromatin, are predicted to show differences allele-specific TF binding affinity, be part of regions displaying chromatin looping and show chromatin modification characteristic of the presence of a SuperEnhancer.

Given the differences in the prevalence and severity of SLE between different ancestries [32], our strategy was to take advantage of the minor allele frequency differences for risk alleles between ancestries to track down the causal risk alleles at *IKZF1* and *IKZF3*. Through a combination of aligning tag SNPs on European risk haplotypes with the corresponding alleles in non-Europeans and subsequent fine-mapping using the multi-ancestral SLE ImmunoChip dataset, we identified the core risk haplotypes at both loci. At *IKZF1* we successfully reduced the core risk haplotype by ~37% down to 37.7 kb, located 38.5 kb upstream of the transcriptional start site and which includes just 12 tag-SNPs variants for functional annotation, by excluding 174 associated variants.

At *IKZF3*, after haplotype alignments between ancestries, we were still left with 93 tag SNPs over 101 kb in the core risk haplotype. Therefore, the nature of the fine-mapping and subsequent functional annotation was more demanding at this locus. It was therefore necessary to incorporate a trans-ancestral exclusion mapping process to exclude tag SNPs from functional annotation based on their MAF and OR. We did this using the African American samples from the SLE multi-ancestry ImmunoChip, because there is no published SLE GWAS in African American samples. This exclusion strategy was based on the assumption that since SLE is more common in samples of African origin, it was reasonable to assume that European tag-SNPs (MAF_EA_ = 3%), would be more common and exhibit stronger association in SLE cases of African origin. Using this approach, we excluded a total of 66 SNPs (from the 93 tag SNPs) which exhibited MAF_AA_ > MAF_EA_ with MAF > 3%OR_AA_ < OR_EA_, leaving just 27 SNPs over 101 kb for functional annotation.

Therefore, in this manuscript, we set out to discover which of the risk variants at *IKZF1* and *IKZF3* were candidate causal risk alleles for SLE or other immune-related disease. Our results revealed that neither set of risk alleles were cis-eQTLs, nor caused amino acid changes in the Ikaros (encoded by *IKZF1*) or Aiolos (encoded by *IKZF3*) proteins. Consequently, we went on to investigate whether the risk alleles acted via epigenetic mechanisms, such as DNA methylation and DNA hypersensitivity, both of which can influence TF binding and chromatin looping.

Although the utility of DNA methylation in unravelling epigenetic mechanisms is immense, there are only two studies of this heritable, cell-type specific mark in SLE samples, both of which utilized probe-based rather than sequencing-based platforms. The first study revealed significant hypomethylation (correlated with increased gene transcription) at *IKZF3* in CD4+ T cells but not at *IKZF1* [44]. There was no ancestry specific analysis published on this dataset, which may be due to the moderate sample size of each cohort. The second study in Danish SLE samples revealed no evidence of hypermethylation (corresponding to down-regulated gene expression) at *IKZF1* or *IKZF3* in B cells, T cells, monocytes or granulocytes [45]. Determination of a detailed allele-specific methylation map across *IKZF1* and *IKZF3* which takes into account trans-ancestral differences in allele frequencies in SLE awaits sequence-based methylation study in immune cell types from SLE samples of different ancestries during flare and during more quiescent disease.

The data in this manuscript suggest that by far the biggest epigenetic determinant of cell-specific differences in gene regulation at *IKZF1* and *IKZF3* come from measurements of DNAse hypersensitivity. Hotspots delineating regions of open chromatin work provide a permissive landscape to allow allele specific TF binding and chromatin looping. All three types of event contribute to an accessible scaffold for post translational modification of chromatin tails, such as acetylation of lysine 27 on histone 3 (H3K27ac), which delineate enhancer elements.

There is widespread open chromatin in multiple cell types across the risk haplotypes for *IKZF1* in T cell types and in a more diverse set of immune cell types across *IKZF3* (Figure 2 and Figure 3). This made it impossible to prioritize specific risk alleles as being more functionally significant. Similarly, it was not possible to prioritize specific risk alleles which were colocalized with sites of preferential marking by H3K27ac. This is in line with a previous report, which indicated that both *IKZF1* and *IKZF3* contain SuperEnhancers (SE) for multiple immune cell types [43] (Figure A3). These SE groups of enhancers, usually found at master transcription factors, which control the identity of a given cell types. Finally, the chromatin looping observed at *IKZF1* and *IKZF3* bring the risk alleles within the enhancers into closer proximity to promoter elements and make the DNA backbone more accessible to large numbers of additional TFs which characterize SuperEnhancers.

In summary, through a process of layered functional annotation at, using publicly available resources, we have found that the core SLE risk alleles at *IKZF1* and *IKZF3* are part of “functionally active DNA,” within SuperEnhancers. Taken together, these results suggest that the *IKZF1* and *IKZF3* risk alleles may contribute to the genetic dysregulation of the SuperEnhancers and the consequential dysregulation in the function of immune cell types. However, we accept that confirmation of these findings requires detailed “wet lab” experimentation, which is outside the remit of this current manuscript.

## 4. Materials and Methods

### 4.1. Datasets

We used 1000-Genome imputed GWAS data from the European GWAS [12] and the two Chinese GWAS [7,31]. The entire 1000-Genome imputed SLE ImmunoChip data from Europeans (n_cases_ = 6748, n_controls_ = 11,516) and African Americans (AA) (n_cases_ = 2970, n_controls_ = 2452) was available through collaboration [33]. The 1000 Genomes data for the five super-populations was downloaded from the 1000 Genomes website via Ensembl. All the genetic data were aligned using the UCSC hg19 build.

### 4.2. Haplotype Analysis of the Genetic Datasets

Haplotypes were derived in each dataset, using the Solid-Spine algorithm in Haploview, (HWE cut off of 0.0001 and minor allele frequency cut off of 0.01) [46]. Visual inspection of overlapping haplotype blocks in the European SLE GWAS was used to identify continuous risk haplotypes across *IKZF1* and *IKZF3*, using an inter-block D′ score of > 0.75 and to select sets of tag SNPs. The European risk alleles and haplotypes were used as a template to align the haplotypes from the other datasets and to track the presence of the European risk haplotype in these populations. The core risk haplotypes were defined by minimal alignment of the haplotype blocks from each dataset.

### 4.3. Trans-Ancestral Meta-Analysis

Trans-ancestral meta-analysis was undertaken using PLINK with the default settings for combining two datasets using a random effect and a fixed effects model [47]. A test of heterogeneity was used to confirm that the datasets were homogenous using a *p* value cut off of >0.01.

### 4.4. Trans-Ancestral Exclusion Mapping

Trans-ancestral exclusion mapping was carried out at *IKZF3* using the EUR (n_cases_ = 6748, n_controls_ = 11,516) and AA (n_cases_ = 2970, n_controls_ = 2452) samples from the SLE ImmunoChip dataset and the EUR and AFR samples from the 1000 Genomes data. Variants were included in the analysis if >75% individuals were typed in each study. The SNPs were aligned by genomic position across all four studies, recording minor allele frequency (MAF) and/or association *p* value/OR for each variant. SNPs were grouped by the differences in MAF between EA/EUR and AA/AFR samples, taking into account the association *p* value where available. A set of European risk alleles which were most likely to tag the causal alleles at *IKZF3* in Europeans were defined as being absent/very rare (MAF < 0.01) in Africans.

### 4.5. Functional Annotation of Risk Alleles

***The H3K27ac epigenetic data*** for the core association intervals and flanking regions (<10kb) was downloaded from the RoadMap Consortium in a total of 27 blood cell-types together with three fibroblast cell-types and a lung endothelial cell-type for use as a control. The epigenetic data contained the consolidated imputed epigenetic data based on the *p* value signals from each of the individual epigenetic marks in each of the cell-types. We used the UCSC genome browser (hg19) to subset each epigenetic track for the required intervals and then exported the signal data via Galaxy [48]. Where the SNPs of interest were <10 bp away from the edge of the 25-bp epigenetic interval containing it, we averaged the enrichment from two adjacent intervals. The Signal Values for the ***DNAse I Hotspot*** data from ENCODE/Washington were downloaded for each of the risk alleles at *IKZF1* and *IKZF3* using UCSC/Galaxy. We accessed the ***PC Hi-C*** data across *IKZF1* and *IKZF3* in immune cell types from the 3D Genome Browser [39,49]. The ***Combined Genome Segmentation data*** from ENCODE in EBV-LCLs was extracted from the UCSC Genome Browser [50]. We used the R package haploR to extract ***cis-eQTL data*** for risk alleles across *IKZF1* and *IKZF3* from Haploreg [30,51] and accessed conditional cis-eQTLs across both genes from the NESDR NTR conditional eQTL database [38]. We exported the enhancers intervals inferred across *IKZF1* and *IKZF3* from the GeneHancer database [35].

### 4.6. Allele-Specific Transcription Factor Binding

For each allele of the tag-SNPs on the core associated haplotypes for *IKZF1* and *IKZF3*, we extracted the predicted allele-specific differences in binding affinity of transcription factor from Haploreg v4.1 using haploR [51]. These differences were calculated as the change in log-odds (LOD) score between the Ref and Alt alleles for each tag-SNP—using Position Weight Matrices (PWM) for any TF binding motifs overlapping a 29 bp region around each risk allele, which reached a stringency (threshold of *P*  <  4^−8^) for either the Ref or Alt allele [30].

### 4.7. Visualisation of Genomic Data

We visualized the epigenetic and genomic data within the UCSC genome browser or using the Gviz package from Bioconductor, within R [52].

## Figures and Tables

**Table 1:** Allele Specific Binding of Transcription Factors to *IKZF1* Risk alleles.

Enhancer Region (Enh)				Promoter/TSS Region (PC Hi-C)			GeneHancer Promoter Region (GH07J050293)		
Risk SNP	TF Showing Allele-Specific Binding (ASTF)		Alt-Ref Enrichment	TSS SNP with Same TF Binding Site as Risk Allele	TF Binding to TSS SNP	Alt-Ref Enrichment	TSS SNP with Same TF Binding Site as Risk Allele	TF Binding to TSS SNP	Alt-Ref Enrichment
rs11185603 *	**A**	**RXRA_disc4**	−11.1	rs146295095	RXRA_known1	3			
				rs141865623	RXRA_disc2	−0.8			
				rs11765436 #	RXRA_disc2	5.7	rs11765436 #	RXRA_disc2	5.7
				rs187496825	RXRA_known2	12			
				rs180969166 ^	RXRA_known6	0			
				rs183264036 ^	RXRA_disc1	0.2	rs7802443 #	RXRA_disc2	11.4
	**B**	**PU.1_disc3**	−11.9	rs191336126 rs80161560	PU.1_disc2	0.8	rs9886239 *	PU.1_disc2	−12
					PU.1_disc3	1.6			
	**C**	**TATA_disc7**	−6.3	rs142010565	TATA_known1	−1.9	rs7777365	TATA_known3	−2.4
				rs142762599	TATA_known1	0.1			
				rs79391891	TATA_disc2	−12			
				rs186224998	TATA_disc9	−4			
				rs62447182	TATA_disc9	−5.1			
**rs876036**	**D**	**ERalpha-a_disc4**	10.5	rs180969166 ^ rs183264036 rs151114892 rs145086785	ERalpha-a_disc2/4	−2/−0.3			
					ERalpha-a_disc4	3.3			
					ERalpha-a_disc4	−3.6			
					ERalpha-a_disc4	0.5			
	**D**	**VDR_2/3**	−7.8, −3.9	rs180969166 ^	VDR_4	12			
				rs151114892	VDR_4	−11.5			
	**E**	**RXRA_known4**	−10.6				rs11765436 #	RXRA_disc2	5.7
							rs7802443 #	RXRA_disc2	11.4
rs876038 *	**A**	**XBP-1_1**	−12	rs184933329	XBP-1_2	−11.9			
				rs74607523	XBP-1_2	−2.3			
	**B**	**BDP1_disc1**	−0.6				rs11761922 *	BDP1_disc1	12
							rs7781977 #	BDP1_disc1	12
	**C**	**Brachyury_1**	−3.2				rs10269380 *	Brachyury_1	4.8
**rs876039**		**Foxa_known2,3**	1.1, 0.6				rs7777365 #	Foxa_known1	−2.7

*/# Risk variants having shared TF binding sites with promoter variants which are eQTLs for *IKZF1* in whole blood (GTEx2015_v6* or NESDA NTR conditional eQTL database#). ^ SNP is just outside PC Hi-C interaction region but within GeneHancer promoter interaction region.

**Table 2:** Allele-Specific Binding of Transcription Factors to Group 1 Risk Alleles at *IKZF3.*

		Group I Risk Variants			*SNPs in IKZF3*-*ZPBP2* Bi-Directional Promoter		
	Risk SNP	Interaction Fragment	ASTF *	Alt-Ref Enrichment	Promoter SNP	Shared Promoter TF	Alt-Ref Enrichment
**1**	**rs111678394**	*IKZF3*-*ZPBP2*	Foxi1	−3.9	-	-	-
			Foxo_1	−2.1	rs138959946 ^a^	Foxo1	−2.4
			Pax-4_5	−2.3	rs189743120 ^a^	Pax_4_5	1
**2**	**rs117278702**	*IKZF3*-*ZPBP2*	-	-	-	-	-
**3**	**rs77924338**	no	VDR_4	−9.1	rs74805134 ^b^	VDR_2	−11.5
**4**	**rs113233720**	no	DMRT4	−11.5	rs147630723 ^a^	DMRT4	11.9
**5**	**rs112677036**	no	Mef2_known5	11.5	rs73985223 ^b^	Mef2_known6	11.9
					rs73985223 ^b^	Mef2_disc1	6.4
					rs4622539 ^b^	Mef2_known5	−3.2
					rs192412458 ^a^	Mef2_disc3	11.9
					rs188089973	Mef2_known5	−3.8
					rs185330833 ^a^	Mef2_known6	11.7
					rs184966935 ^a^	Mef2_known1	−10
					rs184525456 ^a^	Mef2_known5	−3.1
					rs140511615 ^a^	Mef2_known5	−11.8
**6**	**rs111691913**	no	Zntb3	8.0	-	-	-
**7**	**rs111944912**	no	Hoxa13	2	rs12150079	Hoxa13	0.7
**8**	**rs111734595**	no	-	-	-	-	-
**9**	**rs113479772**	no	-	-	-	-	-
**10**	**rs112797570**	no	-	-	-	-	-
**11**	**rs111734595**	no	SETDB1 Zfx	8.2 −5.7	rs201229892 rs117064469	SETDB1 Zfx	−0.6 −1.4
**12**	**rs111469562**	no	Obox6	4.6	rs11078925	Obox3	−6.7
			Dmbx1	4.1	rs11078925	Dmbx1	−9
**13**	**rs112743130**	5′ (I3)	-	-	-	-	-
**14**	**rs112412105**	5′ (I3)	GR_disc4	−12	rs183478341 ^u/k^	GR_disc1	6.6
**15**	**rs113115305**	3′ (E4-7)	-	-	-	-	-
**16**	**rs112238900**	3′ (E4-7)	-	-	-	-	-
**17**	**rs113064843**	3′ (E4-7)	-	-	-	-	-
**18**	**rs16965347**	3′ (E4-7)	Pou6f1_2		-	-	-
**19**	**rs113369293**	3′ (E4-7)	Irf_disc3	2.3	rs138461720 ^u/k^	Irf_disc3	5.5
			Irf_disc3	2.3	rs112745149 ^u/k^	Irf_disc3	9.6
**20**	**rs75148376**	3′ (E4-7)	Ncx_2	4	rs9905881 ^b^	Ncx_2	3.2
			Nkx6-1_2	6.7	rs149800216 ^a^	Nkx6-1_3	−9.7
			Nkx6-1_2	6.7	rs149800216 ^a^	Nkx6-1_2	−10.2
			Nkx6-1_2	6.7	rs149800216 ^a^	Nkx6-1_1	−12
			Ncx_2	4	rs149800216 ^a^	Ncx_2	−6.4
			Pou4f3	5.6	rs138350717 ^a^	Pou4f3	5.9
			Nkx6-1_2	6.7	rs138350717 ^a^	Nkx6-1_2	3.5
			Nkx6-1_2	6.7	rs138350717 ^a^	Nkx6-1_1	7.9
			Dbx1	2.2	rs202227901 ^b^	Dbx1	−0.1
			Dbx1	2.2	rs138350717 ^a^	Dbx1	0.6
			Dbx1	2.2	rs145735506 ^a^	Dbx1	1.4
			Dbx1	2.2	rs185330833 ^a^	Dbx1	−1.2
			Hoxb4	2.1	rs202227901 ^b^	Hoxb4	−0.5
**21**	**rs112771646**	3′ (E4-7)	GR_disc5	−3.8	rs192800564 ^a^	GR_disc6	−9.2
			GR_disc5	−3.8	rs192412458 ^a^	GR_disc2	11.8
			GR_disc5	−3.8	rs11655198	GR_disc4	12
**22**	**rs112301322**	3′ (E4-7)	NF-E2_disc1	11.9	rs201229892 ^a^	NF-E2_disc1	12
			Rad21_disc10	−11.5	rs187549822 ^a^	Rad21_disc2	−4.2
**23**	**rs111862642**	3′ (E4-7)	Sin3Ak-20_disc1	−2.9	rs116467677 ^a^	Sin3Ak-20_disc6	−0.6
**24**	**rs112345383**	3′ (E4-7)	HNF1_2	6.1	rs202236981 ^a^	HNF1_2	−1.8
**25**	**rs113370572 ^T^**	3′ (E4-7)	HDAC2_disc5	9.6	rs202227901 ^b^	HDAC2_disc6	10.6
			HDAC2_disc5	9.6	rs200781948 ^a^	HDAC2_disc6	−3.9
**26**	**rs112771360**	no	-	-	-	-	-
**27**	**rs112876941**	no	HNF1_7	3.5	-	-	-
			HNF1_6	3.1	rs9905881 ^b^	HNF1_6	−2.7
			HNF1_6	3.1	rs9907564 ^b^	HNF1_6	−1.1
			HNF1_6	3.1	rs138350717 ^a^	HNF1_6	0.7
			HNF1_1	4.3	rs9905881 ^b^	HNF1_1	−4.3
			Foxo_2	11.9	rs184525456 ^a^	Foxo_2	−12
			Foxa_disc2	−10.6	rs145895912 ^a^	Foxa_disc3	11.7
			Foxj1_1	4.6	rs145735506 ^a^	Foxj1_1	11.8
			Foxo_2	11.9	rs138959946 ^a^	Foxo_2	−12

* ASTFs predicted to exhibit >2 fold enrichment when binding to Group 1 risk allele compared with binding to the non-risk allele; ^T^ Group 1 SNP in TSS (~8.4 kb) of shorter isoform; For promoter variants: ^a^ very rare minor allele (<0.5% or monomorphic) in EUR; **^b^** ~3% minor allele in EUR u/k—within promoter interaction region not within risk haplotype.

## Abbreviations

Tag-SNPs	Single nucleotide polymorphism tagging a haplotype
SLE GWAS	Genome-Wide association study in Systemic Lupus Erythematosus
ASTF	Allele-Specific transcription factor binding site
eQTL	Expression quantitative trait locus
GWAS	Genome-Wide association study
MAF	Minor allele frequency
PC Hi-C	Promoter Capture Hi-C
SLE	Systemic Lupus Erythematosus
TF	Transcription factor

## Appendix A

**Table A1:** Association at *IKZF1* in Trans-ancestral SLE ImmunoChip Study.

SNP	Pos (hg19)	African American *2970 Cases, 2452 Controls*			European *6748 Cases, 11,516 Controls*			Hispanic *1872 Cases and 2016 Controls*		
		*p* Value	OR_AA_ (CI)	MAF_AA_	*p* Value	OR_EA_ (CI)	MAF_EA_	*p* Value	OR_Hisp_ (CI)	MAF_Hisp_
**rs4917014**	**7:50305863**	1.48 × 10^−5^	0.728 (0.631–0.841)	0.09 (G)	3.67 × 10^−9^	0.866 (0.826–0.909)	0.32 (G)	0.021	0.897 (0.818–0.984)	0.48 (T)
**rs11185603**	**7:50306810**	4.29 × 10^−5^	0.742 (0.643–0.856)	0.09 (G)	8.99 × 10^−9^	0.870 (0.829–0.912)	0.32 (G)	0.021	0.898 (0.819–0.984)	0.48 (C)
**rs4385425**	**7:50307334**	1.83 × 10^−5^	0.831 (0.771–0.897)	0.49 (G)	1.51 × 10^−9^	0.872 (0.832–0.914)	0.32 (G)	0.148	0.934 (0.852–1.026)	0.50 (A)
**rs876036**	**7:50307710**	9.52 × 10^−3^	0.890 (0.815–0.972)	0.25 (C)	7.49 × 10^−9^	0.869 (0.829–0.912)	0.32 (C)	0.053	0.913 (0.833–1.001)	0.49 (T)
**rs876037**	**7:50308692**	1.87 × 10^−5^	0.731 (0.633–0.844)	0.09 (A)	2.23 × 10^−8^	0.873 (0.832–0.915)	0.31 (A)	0.020	0.897 (0.818–0.983)	0.48 (T)

**Table A2:** Genomic Locations of Regulatory Elements at *IKZF1* and *IKZF3.*

Locus	Element	Name	Position (hg19)
***IKZF1***	**PC Hi-C interaction regions**	Enhancer (Enh)	chr7:50305428-50311993
		Transcriptional Start Site/Promoter (TSS)	chr7:50341186-50347256
		intron 3 (I3)	chr7:50411807-50412756
	**GeneHancer regions**	GH07J050261	chr7:50300992-50310765
		GH07J050293	chr7:50333047-50334464
		GH07J050301	chr7:50340632-50340761
		GH07J050303	chr7:50343395-50362927
		GH07J050326	chr7:50366368-50368325
		GH07J050329	chr7:50368690-50370631
		GH07J050341	chr7:50410631-50437890
		GH07J050392	chr7:50459865-50466852
***IKZF3***	**PC Hi-C interaction regions**	*IKZF3*-*ZPBP2* bi-directional promoter	chr17:38018444-38027003
		5′ I3	chr17:37965773-37976506
		mid I3	chr17:37958027-37963133
		3′ E4-7	chr17:37932293-37957717
	**GeneHancer regions**	GH17J039753	chr17:37909296-37916397
		GH17J039766	chr17:37922530-37939749
		GH17J039790	chr17:37946728-37952847
		GH17J039799	chr17:37954622-37954701
		GH17J039798	chr17:37954998-37957986
		GH17J039812	chr17:37968642-37971311
		GH17J039817	chr17:37974070-37978821
		GH17J039839	chr17:37995815-37995875
		GH17J039842	chr17:37999223-38000547
		GH17J039847	chr17:38003768-38005630

**Table A3:** Allele-Specific Binding of Transcription Factors to *IKZF1* Risk Alleles.

Order	Risk SNP	Pos (hg19)	TF Showing Allele-Specific Binding (ASTF)	Strand	Ref	Alt	Alt-Ref Enrichment
**1**	**rs34767118**	**50271064**	Sox_5	+	12.5	11.4	−1.1
			VDR_1	+	−8.1	3.9	12
			Zbtb12	+	11.8	14.4	2.6
**2**	**rs11773763**	**50271499**	CDP_4	-	12.6	13.2	0.6
			Fox	-	13.3	2.5	−10.8
			Foxd1_1	-	4.5	2.5	−2
			Foxi1	-	13.1	11.9	−1.2
			Foxj1_2	-	14.2	13.9	−0.3
			Foxj2_1	-	12.1	12	−0.1
			Gm397	-	6.6	10.7	4.1
			Pou3f2_2	+	−9.4	2.6	12
			Zfp105	+	10.8	11	0.2
			p53_1	+	−25.8	−27.5	−1.7
**3**	**rs62445350**	**50278187**	none				0
**4**	**rs62445352**	**50289504**	Arid3a_2	-	8.4	10.9	2.5
			Barx2	-	10.5	11.9	1.4
			Cdx2_2	-	10.6	11.2	0.6
			Dbx1	-	8.7	10.6	1.9
			Dbx2	+	8.8	11.5	2.7
			Dlx3	-	12.1	10	−2.1
			Evi-1_4	+	4	15.5	11.5
			HNF1_1	-	12.7	10.7	−2
			HNF1_6	-	13.8	11.2	−2.6
			HNF1_7	+	11.7	10.2	−1.5
			Hoxa10	-	11	12.8	1.8
			Hoxa3_2	-	13.9	13.1	−0.8
			Hoxa5_3	-	11.6	10.2	−1.4
			Hoxa7_2	-	11	12.5	1.5
			Hoxb4	-	11.2	12.4	1.2
			Hoxc6	-	12.1	13	0.9
			Hoxc9	-	12.2	12.7	0.5
			Hoxd8	+	12.9	16.1	3.2
			Msx-1_2	-	10.7	13.2	2.5
			Ncx_2	-	11.4	15.1	3.7
			Nkx6-1_2	-	9.9	14.8	4.9
			Nkx6-1_3	-	9.7	14.9	5.2
			Nkx6-2	-	11.6	12.6	1
			Pax-4_2	-	11.2	8.1	−3.1
			Pou2f2_known4	+	12.8	13.3	0.5
			Pou3f4	-	6	11.7	5.7
			Pou4f3	-	9.1	15.1	6
			Pou5f1_known1	+	11.6	4.7	−6.9
			Prrx1	+	11	10.4	−0.6
**5**	**rs55935382**	**50289669**	SRF_known5	+	−0.8	11	11.8
**6**	**rs11185602**	**50299077**	Cart1	+	15.2	11.7	−3.5
			Cdx	+	9.6	12.1	2.5
			HNF1_2	-	6.2	11.3	5.1
			Lhx3_2	+	10.7	3	−7.7
			PLZF	+	13.2	13	−0.2
			Pou2f2_known2	+	12.8	8.4	−4.4
			Pou2f2_known9	+	7.4	−4.5	−11.9
			Pou6f1_1	-	10.2	13.9	3.7
**7**	**rs4917014 ***	**50305863**	Nkx2_2	+	10.9	12	1.1
**8**	**rs11185603 ***	**50306810**	CCNT2_disc2	+	12.5	7.1	−5.4
			ELF1_known1	-	13	2	−11
			Nkx2_2	-	11.9	10.3	−1.6
			PU.1_disc3	-	12.3	0.4	−11.9
			RXRA_disc4	+	12.8	1.7	−11.1
			TATA_disc7	-	13.6	7.3	−6.3
**9**	**rs4385425 ***	**50307334**	none				0
**10**	**rs876036 ***	**50307710**	ERalpha-a_disc4	+	0.2	10.7	10.5
			LXR_3	-	11.3	7.4	−3.9
			RXRA_known4	+	10.4	−0.2	−10.6
			VDR_2	+	12.4	4.6	−7.8
			VDR_3	+	12.2	8.3	−3.9
**11**	**rs876038 ***	**50308527**	BDP1_disc1	-	2.7	2.1	−0.6
			Brachyury_1	-	−2.4	−5.6	−3.2
			XBP-1_1	+	12.2	0.2	−12
**12**	**rs876037 ***	**50308527**	none				0
**13**	**rs876039 ***	**50308811**	Foxa_known2	-	11.5	12.6	1.1
			Foxa_known3	-	12.7	13.3	0.6

* SLE risk variants lying within the *IKZF1* GeneHancer enhancer (GH07J050261).

**Table A4:** Meta-Analysis of EA Tagging SNPs across *IKZF3* in ImmunoChip data from European and African Ancestries.

#	Group	rs	Chr	Pos (hg19)	A1/A2	ImmunoChip Association Data						Meta-Analysis					
						MAF_EA_	*P*_EA_	OR_EA_	MAF_EA_	P_AA_	OR_AA_	*s*	*P*(R)	OR	OR(R)	Q	I
**1**	**1**	rs111678394	17	38021116	C/G	0.035	2.50 × 10^−6^	1.29 (1.16–1.44)	0.005	0.042	1.656 (1.01–2.71)	5.29 × 10^−7^	5.29 × 10^−7^	1.31	1.31	0.335	0
**2**	**1**	rs117278702	17	38020420	A/G	0.032	1.13 × 10^−5^	1.28 (1.15–1.44)	0.004	0.136	1.50 (0.877–2.55)	4.38 × 10^−6^	4.38 × 10^−6^	1.30	1.30	0.588	0
**3**	**2**	rs9905881	17	38018954	A/G	0.036	4.44 × 10^−6^	1.28 (1.15–1.43)	0.256	0.004	1.13 (1.04–1.24)	3.16 × 10^−7^	0.003	1.19	1.20	0.079	67.8
**4**	**2**	rs9899336	17	38017779	T/C	0.036	3.60 × 10^−6^	1.28 (1.16–1.43)	0.256	0.005	1.13 (1.04–1.23)	3.50 × 10^−7^	0.004	1.19	1.20	0.066	70.5
**5**	**2**	rs9899006	17	38017064	A/T	0.042	1.28 × 10^−5^	1.25 (1.13–1.38)	0.257	0.005	1.13 (1.04–1.23)	7.23 × 10^−7^	0.001	1.18	1.18	0.137	54.8
**6**	**1**	rs77924338	17	38016356	T/C	0.035	2.50 × 10^−6^	1.29 (1.16–1.44)	0.005	0.042	1.66 (1.01–2.71)	5.29 × 10^−7^	5.29 × 10^−7^	1.31	1.31	0.335	0
**7**	**2**	rs9915797	17	38014867	A/G	0.036	2.75 × 10^−6^	1.29 (1.16–1.44)	0.256	0.005	1.13 (1.04–1.23)	3.40 × 10^−7^	0.005	1.19	1.20	0.056	72.6
**8**	**2**	rs16965367	17	38014315	C/T	0.036	3.99 × 10^−6^	1.28 (1.15–1.43)	0.256	0.005	1.13 (1.04–1.23)	3.68 × 10^−7^	0.004	1.19	1.20	0.069	69.9
**9**	**2**	rs113466546	17	38012586	A/G	0.036	2.10 × 10^−6^	1.29 (1.16–1.44)	0.130	0.026	1.13 (1.02–1.27)	7.90 × 10^−7^	0.004	1.21	1.21	0.090	65.2
**10**	**2**	rs9907291	17	38010036	G/A	0.036	2.75 × 10^−6^	1.29 (1.16–1.44)	0.257	0.003	1.14 (1.05–1.24)	1.57 × 10^−7^	0.003	1.20	1.21	0.072	69.1
**11**	**2**	rs8069531	17	38009343	T/A	0.036	3.24 × 10^−6^	1.29 (1.16–1.43)	0.256	0.005	1.13 (1.04–1.23)	3.36 × 10^−7^	0.004	1.19	1.21	0.064	70.9
**12**	**2**	rs8068894	17	38008999	G/A	0.036	2.75 × 10^−6^	1.29 (1.16–1.44)	0.256	0.005	1.13 (1.04–1.23)	2.87 × 10^−7^	0.005	1.19	1.20	0.060	71.9
**13**	**1**	rs113233720	17	38008190	T/C	0.035	2.50 × 10^−6^	1.29 (1.16–1.44)	0.005	0.042	1.66 (1.01–2.71)	5.29 × 10^−7^	5.29 × 10^−7^	1.31	1.31	0.335	0
**14**	**1**	rs112677036	17	38002152	A/G	0.035	2.50 × 10^−6^	1.29 (1.16–1.44)	0.005	0.042	1.66 (1.01–2.71)	5.29 × 10^−7^	5.29 × 10^−7^	1.31	1.31	0.335	0
**15**	**2**	rs67600807	17	38001558	G/A	0.036	2.75 × 10^−6^	1.29 (1.16–1.44)	0.262	0.007	1.13 (1.03–1.22)	4.54 × 10^−7^	0.008	1.19	1.20	0.049	74.2
**16**	**2**	rs9908694	17	37997771	T/C	0.036	2.90 × 10^−6^	1.29 (1.16–1.43)	0.256	0.005	1.13 (1.04–1.23)	2.95 × 10^−7^	0.004	1.19	1.20	0.061	71.6
**17**	**2**	rs9900541	17	37996070	C/T	0.036	2.75 × 10^−6^	1.29 (1.16–1.44)	0.256	0.005	1.13 (1.04–1.23)	3.12 × 10^−7^	0.005	1.19	1.20	0.058	72.3
**18**	**1**	rs111691913	17	37993238	T/C	0.035	2.24 × 10^−6^	1.30 (1.16–1.44)	0.005	0.042	1.66 (1.01–2.71)	4.60 × 10^−7^	4.60 × 10^−7^	1.31	1.31	0.338	0
**19**	**2**	rs28449671	17	37991630	C/T	0.036	2.47 × 10^−6^	1.29 (1.16–1.44)	0.256	0.005	1.13 (1.036–1.23)	3.27 × 10^−7^	0.006	1.19	1.20	0.055	73.0
**20**	**1**	rs111944912	17	37988476	C/T	0.035	3.64 × 10^−6^	1.29 (1.16–1.43)	0.005	0.042	1.66 (1.01–2.71)	7.80 × 10^−7^	7.80 × 10^−7^	1.30	1.30	0.326	0
**21**	**2**	rs73304123	17	37987588	T/C	0.036	3.07 × 10^−6^	1.29 (1.16–1.43)	0.128	0.025	1.14 (1.02–1.27)	9.54 × 10^−7^	0.003	1.21	1.21	0.108	61.4
**22**	**2**	rs112141468	17	37987464	T/C	0.036	3.99 × 10^−6^	1.28 (1.15–1.43)	0.259	0.006	1.13 (1.04–1.23)	4.64 × 10^−7^	0.005	1.19	1.20	0.063	71.2
**23**	**1**	rs111734595	17	37987399	T/C	0.035	3.64 × 10^−6^	1.29 (1.16–1.43)	0.005	0.042	1.66 (1.01–2.71)	7.80 × 10^−7^	7.80 × 10^−7^	1.30	1.30	0.326	0
**24**	**1**	rs113479772	17	37987042	A/G	0.035	3.64 × 10^−6^	1.29 (1.16–1.43)	0.005	0.042	1.66 (1.01–2.71)	7.80 × 10^−7^	7.80 × 10^−7^	1.30	1.30	0.326	0
**25**	**1**	rs112797570	17	37983751	A/G	0.035	3.64 × 10^−6^	1.29 (1.16–1.43)	0.005	0.042	1.66 (1.01–2.71)	7.80 × 10^−7^	7.80 × 10^−7^	1.30	1.30	0.326	0
**26**	**1**	rs112437508	17	37983512	A/G	0.035	3.09 × 10^−6^	1.29 (1.16–1.44)	0.023	0.564	1.07 (0.840–1.38)	6.90 × 10^−6^	0.016	1.25	1.22	0.190	41.7
**27**	**2**	rs35130019	17	37983141	G/A	0.037	6.29 × 10^−6^	1.28 (1.15–1.42)	0.255	0.007	1.13 (1.03–1.23)	7.56 × 10^−7^	0.004	1.18	1.19	0.073	68.8
**28**	**1**	rs111469562	17	37982696	C/T	0.036	4.51 − 0^6^	1.28 (1.15–1.43)	0.005	0.042	1.66 (1.01–2.71)	9.58 × 10^−7^	9.58 × 10^−7^	1.30	1.30	0.321	0
**29**	**2**	rs12942660	17	37982037	T/C	0.036	4.44 × 10^−6^	1.28 (1.15–1.43)	0.252	0.003	1.14 (1.04–1.24)	2.54 × 10^−7^	0.002	1.19	1.20	0.085	66.2
**30**	**2**	rs8076347	17	37977540	T/G	0.036	3.78 × 10^−6^	1.28 (1.16–1.42)	0.252	0.003	1.14 (1.04–1.24)	2.29 × 10^−7^	0.002	1.19	1.20	0.081	67.1
**31**	**2**	rs9908983	17	37976926	A/G	0.036	3.42 × 10^−6^	1.29 (1.16–1.43)	0.124	0.023	1.14 (1.02–1.27)	8.39 × 10^−7^	0.002	1.21	1.21	0.122	58.2
**32**	**2**	rs9911069	17	37976601	C/T	0.036	3.07 × 10^−6^	1.29 (1.16–1.43)	0.124	0.023	1.14 (1.02–1.27)	7.99 × 10^−7^	0.002	1.22	1.21	0.120	58.7
**33**	**2**	rs9901917	17	37976205	C/G	0.036	3.42 × 10^−6^	1.29 (1.16–1.43)	0.124	0.023	1.14 (1.02–1.27)	8.39 × 10^−7^	0.002	1.21	1.21	0.122	58.2
**34**	**1**	rs112743130	17	37975855	C/G	0.035	3.46 × 10^−6^	1.29 (1.16–1.43)	0.005	0.059	1.59 (0.979–2.58)	8.55 × 10^−7^	8.55 × 10^−7^	1.30	1.30	0.406	0
**35**	**2**	rs34053394	17	37975660	G/A	0.036	3.42 × 10^−6^	1.29 (1.16–1.43)	0.124	0.023	1.13 (1.02–1.27)	8.39 × 10^−7^	0.002	1.21	1.21	0.122	58.2
**36**	**2**	rs58075375	17	37975592	T/C	0.036	3.42 × 10^−6^	1.287 (1.157–1.432)	0.124	0.023	1.14 (1.02–1.27)	8.39 × 10^−7^	0.002	1.21	1.21	0.122	58.2
**37**	**2**	rs9902621	17	37973010	A/G	0.036	3.42 × 10^−6^	1.29 (1.16–1.43)	0.124	0.023	1.14 (1.02–1.27)	8.39 × 10^−7^	0.002	1.21	1.21	0.122	58.2
**38**	**2**	rs9898031	17	37972647	G/C	0.036	6.45 × 10^−6^	1.28 (1.16–1.42)	0.124	0.023	1.14 (1.02–1.27)	1.38 × 10^−6^	0.001	1.21	1.20	0.147	52.4
**39**	**1**	rs112412105	17	37971635	G/A	0.036	4.06 × 10^−6^	1.29 (1.16–1.43)	0.005	0.059	1.59 (0.979–2.58)	9.57 × 10^−7^	9.57 × 10^−7^	1.30	1.30	0.402	0
**40**	**1**	rs113115305	17	37970686	C/A	0.036	9.37 × 10^−6^	1.27 (1.14–1.42)	0.005	0.059	1.59 (0.979–2.58)	2.28 × 10^−6^	2.28 × 10^−6^	1.29	1.29	0.380	0
**41**	**1**	rs112238900	17	37968494	T/C	0.036	4.06 × 10^−6^	1.29 (1.16–1.43)	0.005	0.059	1.59 (0.979–2.58)	9.57 × 10^−7^	9.57 × 10^−7^	1.30	1.30	0.402	0
**42**	**2**	rs67135646	17	37967871	G/C	0.036	5.47 × 10^−6^	1.28 (1.15–1.42)	0.252	0.004	1.13 (1.04–1.24)	4.03 × 10^−7^	0.003	1.19	1.20	0.082	66.9
**43**	**2**	rs114777282	17	37967649	A/C	0.036	5.47 × 10^−6^	1.28 (1.15–1.42)	0.250	0.005	1.13 (1.04–1.23)	4.48 × 10^−7^	0.003	1.19	1.20	0.080	67.3
**44**	**2**	rs4337325	17	37964435	T/C	0.036	9.22 × 10^−6^	1.27 (1.14–1.41)	0.250	0.005	1.13 (1.04–1.23)	7.12 × 10^−7^	0.002	1.18	1.19	0.095	64.2
**45**	**2**	rs9901617	17	37964175	C/G	0.036	4.24 × 10^−6^	1.284 (1.15–1.43)	0.125	0.027	1.13 (1.01–1.27)	1.27 × 10^−6^	0.002	1.21	1.21	0.116	59.6
**46**	**1**	rs113064843	17	37960421	C/T	0.036	5.02 × 10^−6^	1.28 (1.15–1.43)	0.005	0.059	1.59 (0.979–2.58)	1.25 × 10^−6^	1.25 × 10^−6^	1.29	1.29	0.395	0
**47**	**2**	rs7211998	17	37959788	G/A	0.036	6.42 × 10^−6^	1.28 (1.15–1.42)	0.235	0.005	1.13 (1.04–1.23)	5.27 × 10^−7^	0.002	1.19	1.20	0.089	65.4
**48**	**2**	rs36097841	17	37958112	A/G	0.036	6.08 × 10^−6^	1.28 (1.15–1.42)	0.252	0.002	1.14 (1.05–1.24)	2.69 × 10^−7^	0.001	1.19	1.20	0.101	62.8
**49**	**2**	rs34988504	17	37957631	T/C	0.036	5.47 × 10^−6^	1.28 (1.15–1.42)	0.252	0.004	1.14 (1.04–1.24)	3.14 × 10^−7^	0.002	1.19	1.20	0.089	65.4
**50**	**1**	rs16965347	17	37957566	C/G	0.030	1.24 × 10^−5^	1.29 (1.15–1.45)	0.004	0.154	1.52 (0.852–2.70)	5.12 × 10^−6^	5.12 × 10^−6^	1.30	1.30	0.595	0
**51**	**2**	rs12937330	17	37957316	A/C	0.036	6.02 × 10^−6^	1.28 (1.15–1.42)	0.268	0.009	1.12 (1.03–1.22)	1.33 × 10^−6^	0.008	1.18	1.19	0.056	72.7
**52**	**2**	rs34344462	17	37955193	G/A	0.036	5.47 × 10^−6^	1.28 (1.15–1.42)	0.252	0.005	1.13 (1.04–1.23)	4.78 × 10^−7^	0.003	1.19	1.20	0.078	67.82
**53**	**2**	rs9899345	17	37954757	A/G	0.035	2.37 × 10^−5^	1.26 (1.13–1.41)	0.251	0.004	1.13 (1.04–1.24)	1.17 × 10^−6^	0.001	1.18	1.20	0.125	57.4
**54**	**1**	rs113369293	17	37952654	T/C	0.036	4.51 × 10^−6^	1.28 (1.15–1.43)	0.007	0.287	1.27 (0.820–1.95)	2.63 × 10^−6^	2.63 × 10^−6^	1.28	1.28	0.948	0
**55**	**1**	rs75148376	17	37952508	T/C	0.036	4.51 × 10^−6^	1.28 (1.15–1.43)	0.007	0.287	1.27 (0.820–1.95)	2.63 × 10^−6^	2.63 × 10^−6^	1.28	1.28	0.948	0
**56**	**2**	rs73302152	17	37952350	C/G	0.036	6.84 × 10^−6^	1.28 (1.15–1.42)	0.127	0.059	1.11 (0.996–1.25)	5.54 × 10^−6^	0.010	1.20	1.19	0.081	67.1
**57**	**2**	rs113159227	17	37952091	A/G	0.036	3.81 × 10^−6^	1.29 (1.16–1.43)	0.127	0.063	1.11 (0.994–1.24)	4.04 × 10^−6^	0.014	1.20	1.20	0.065	70.7
**58**	**2**	rs56928975	17	37952031	G/A	0.048	2.38 × 10^−7^	1.28 (1.16–1.40)	0.250	0.014	1.11 (1.02–1.22)	1.18 × 10^−7^	0.011	1.19	1.19	0.034	77.7
**59**	**2**	rs12938749	17	37951847	T/C	0.036	3.81 × 10^−6^	1.29 (1.16–1.43)	0.127	0.063	1.11 (0.994–1.24)	4.04 × 10^−6^	0.014	1.20	1.20	0.064	70.7
**60**	**2**	rs35938199	17	37950812	T/C	0.036	4.24 × 10^−6^	1.28 (1.15–1.43)	0.127	0.063	1.11 (0.994–1.24)	4.23 × 10^−6^	0.014	1.20	1.20	0.066	70.4
**61**	**2**	rs35105110	17	37950421	A/G	0.036	3.24 × 10^−6^	1.29 (1.16–1.43)	0.127	0.063	1.11 (0.994–1.24)	3.63 × 10^−6^	0.014	1.20	1.20	0.062	71.3
**62**	**2**	rs35352075	17	37949790	C/T	0.036	3.81 × 10^−6^	1.29 (1.16–1.43)	0.127	0.063	1.11 (0.994–1.24)	4.04 × 10^−6^	0.014	1.20	1.20	0.064	70.7
**63**	**1**	rs112771646	17	37945708	C/A	0.036	4.51 × 10^−6^	1.28 (1.15–1.43)	0.007	0.287	1.27 (0.820–1.95)	2.63 × 10^−6^	2.63 × 10^−6^	1.28	1.28	0.950	0
**64**	**1**	rs112301322	17	37944518	G/C	0.036	4.51 × 10^−6^	1.28 (1.15–1.43)	0.007	0.287	1.27 (0.820–1.95)	2.63 × 10^−6^	2.63 × 10^−6^	1.28	1.28	0.950	0
**65**	**2**	rs35088469	17	37944481	T/C	0.036	2.21 × 10^−6^	1.29 (1.16–1.44)	0.119	0.096	1.10 (0.983–1.24)	4.27 × 10^−6^	0.024	1.20	1.20	0.048	74.4
**66**	**2**	rs34291217	17	37944410	A/C	0.036	3.81 × 10^−6^	1.29 (1.16–1.43)	0.127	0.063	1.11 (0.994–1.24)	4.04 × 10^−6^	0.014	1.20	1.20	0.065	70.7
**67**	**2**	rs9911688	17	37943800	T/C	0.036	4.71 × 10^−6^	1.28 (1.15–1.43)	0.127	0.060	1.11 (0.996–1.24)	4.20 × 10^−6^	0.011	1.20	1.20	0.073	69.0
**68**	**2**	rs9911669	17	37943766	G/C	0.036	3.81 × 10^−6^	1.29 (1.16–1.43)	0.127	0.063	1.11 (0.994–1.24)	4.04 × 10^−6^	0.014	1.20	1.20	0.065	70.7
**69**	**1**	rs111862642	17	37942983	G/C	0.036	4.51 × 10^−6^	1.28 (1.15–1.43)	0.007	0.287	1.27 (0.820–1.95)	2.63 × 10^−6^	2.63 × 10^−6^	1.28	1.28	0.945	0
**70**	**2**	rs34599546	17	37942971	T/C	0.036	4.93 × 10^−6^	1.28 (1.15–1.42)	0.255	0.010	1.12 (1.03–1.22)	1.10 × 10^−6^	0.008	1.18	1.19	0.055	72.8
**71**	**1**	rs112345383	17	37942017	T/C	0.036	4.51 × 10^−6^	1.28 (1.15–1.43)	0.007	0.287	1.27 (0.82–1.95)	2.63 × 10^−6^	2.63 × 10^−6^	1.28	1.28	0.950	0
**72**	**2**	rs1510475	17	37941379	C/A	0.036	3.81 × 10^−6^	1.29 (1.16–1.43)	0.127	0.063	1.11 (0.994–1.24)	4.04 × 10^−6^	0.014	1.20	1.20	0.065	70.7
**73**	**2**	rs113812449	17	37940167	C/T	0.036	4.93 × 10^−6^	1.28 (1.15–1.42)	0.255	0.010	1.12 (1.03–1.22)	1.10 × 10^−6^	0.008	1.18	1.20	0.055	72.8
**74**	**2**	rs9909365	17	37939958	G/A	0.036	4.93 × 10^−6^	1.28 (1.15–1.42)	0.255	0.010	1.12 (1.03–1.22)	1.10 × 10^−6^	0.008	1.18	1.19	0.055	72.76
**75**	**2**	rs34016964	17	37938976	T/G	0.036	4.93 × 10^−6^	1.28 (1.15–1.42)	0.255	0.010	1.12 (1.03–1.22)	1.10 × 10^−6^	0.008	1.18	1.19	0.055	72.8
**76**	**2**	rs67605703	17	37938496	C/T	0.036	4.24 × 10^−6^	1.28 (1.15–1.43)	0.128	0.078	1.11 (0.989–1.24)	5.78 × 10^−6^	0.019	1.20	1.19	0.057	72.5
**77**	**2**	rs35506518	17	37938093	C/T	0.036	2.61 × 10^−6^	1.29 (1.16–1.44)	0.127	0.060	1.11 (0.996–1.24)	2.70 × 10^−6^	0.01404	1.20	1.20	0.060	71.8
**78**	**2**	rs13380871	17	37936248	C/T	0.036	3.78 × 10^−6^	1.28 (1.16–1.43)	0.255	0.020	1.11 (1.02–1.21)	2.39 × 10^−6^	0.019	1.17	1.19	0.034	77.6
**79**	**2**	rs7224641	17	37934910	C/T	0.036	2.60 × 10^−6^	1.29 (1.16–1.44)	0.255	0.011	1.12 (1.03–1.22)	9.12 × 10^−7^	0.013	1.18	1.20	0.039	76.5
**80**	**2**	rs12709364	17	37933822	G/A	0.036	2.22 × 10^−6^	1.29 (1.16–1.44)	0.128	0.079	1.11 (0.988–1.24)	3.83 × 10^−6^	0.023	1.20	1.20	0.046	74.9
**81**	**1**	rs113370572	17	37933467	C/T	0.035	2.94 × 10^−6^	1.29 (1.16–1.44)	0.007	0.287	1.27 (0.820–1.95)	1.78 × 10^−6^	1.78 × 10-^6^	1.29	1.29	0.932	0
**82**	**2**	rs9901483	17	37932773	A/T	0.036	2.60 × 10^−6^	1.29 (1.16–1.44)	0.255	0.011	1.12 (1.03–1.22)	9.12 × 10^−7^	0.013	1.18	1.20	0.039	76.5
**83**	**2**	rs9894898	17	37932220	C/T	0.036	1.99 × 10^−6^	1.30 (1.16–1.44)	0.127	0.073	1.11 (0.991–1.24)	3.13 × 10^−6^	0.021	1.20	1.20	0.047	74.8
**84**	**2**	rs9913596	17	37932062	A/G	0.036	1.99 × 10^−6^	1.30 (1.16–1.44)	0.127	0.122	1.09 (0.977–1.22)	6.92 × 10^−6^	0.041	1.19	1.20	0.031	78.6
**85**	**2**	rs9652840	17	37929427	T/A	0.037	3.32 × 10^−5^	1.25 (1.13–1.39)	0.201	0.045	1.1 (1.00–1.21)	2.24 × 10^−5^	0.015	1.16	1.17	0.074	68.7
**86**	**2**	rs71369788	17	37927144	A/G	0.036	3.42 × 10^−6^	1.29 (1.16–1.43)	0.200	0.052	1.10 (0.999–1.20)	6.32 × 10^−6^	0.033	1.18	1.19	0.027	79.5
**87**	**2**	rs8072612	17	37927119	G/A	0.036	3.06 × 10^−6^	1.29 (1.16–1.43)	0.255	0.011	1.12 (1.03–1.22)	9.31 × 10^−7^	0.011	1.18	1.20	0.043	75.7
**88**	**2**	rs9894370	17	37926003	C/G	0.037	4.77 × 10^−7^	1.31 (1.18–1.45)	0.318	0.018	1.10 (1.02–1.20)	8.05 × 10^−7^	0.037	1.17	1.20	0.011	84.6
**89**	**2**	rs34758895	17	37925467	T/C	0.037	5.33 × 10-^7^	1.31 (1.18–1.45)	0.318	0.018	1.10 (1.02–1.20)	7.76 × 10-^7^	0.034	1.17	1.20	0.012	84.2
**90**	**1**	rs112771360	17	37923770	G/A	0.035	2.79 × 10^−6^	1.29 (1.16–1.44)	0.007	0.287	1.27 (0.820–1.95)	1.59 × 10^−6^	1.59 × 10^−6^	1.29	1.29	0.926	0
**91**	**1**	rs112876941	17	37922803	T/A	0.035	2.36 × 10^−6^	1.29 (1.16–1.44)	0.007	0.287	1.27 (0.820–1.95)	1.37 × 10^−6^	1.37 × 10^−6^	1.29	1.29	0.921	0
**92**	**2**	rs2941509	17	37921193	T/C	0.037	1.30 × 10^−5^	1.27 (1.14–1.41)	0.244	0.052	1.09 (0.999–1.19)	2.07 × 10^−5^	0.034	1.16	1.17	0.034	77.9
**93**	**2**	rs67571561	17	37920846	C/T	0.036	2.44 × 10^−6^	1.29 (1.16–1.43)	0.230	0.049	1.09 (1–1.20)	6.03 × 10^−6^	0.041	1.17	1.18	0.019	81.8

**Class**: ***Class 1*** MAF_EA_ > MAF_AA_; ***Class 2*** MAF_EA_ < MAF_AA_. **A1/A2**: Risk Allele EA/Non-Risk Allele EA. **ImmunoChip Association data**: MAF, *p* value and OR for EA and AA cohorts. **Meta-analysis**: ***p*** value fixed effects, ***P(R)*** value random effects, ***OR*** fixed effects, ***OR(R)*** Random effects, ***Q*** *p* value for Cochrane’s Q statistic, ***I*** I^2 heterogeneity index (0–100).

**Table A5:** Overlap of *IKZF3* risk alleles with PC Hi-C interaction regions and GeneHancer regulatory elements.

#	Group	rs	Chr	Pos	PC Hi-C Interaction Region	Pos (hg19)	GeneHancer	Pos (hg19)
**1**	**1**	rs111678394	17	38021116	***IKZF3*-*ZPBP2***	**38018444-38027003**	**GH17J039859**	**38015831-38025531**
**2**	**1**	rs117278702	17	38020420				
**3**	**2**	rs9905881	17	38018954				
**4**	**2**	rs9899336	17	38017779				
**5**	**2**	rs9899006	17	38017064				
**6**	**1**	rs77924338	17	38016356				
**7**	**2**	rs9915797	17	38014867				
**8**	**2**	rs16965367	17	38014315				
**9**	**2**	rs113466546	17	38012586				
**10**	**2**	rs9907291	17	38010036				
**11**	**2**	rs8069531	17	38009343			**GH17J039852**	**38008382-38009513**
**12**	**2**	rs8068894	17	38008999				
**13**	**1**	rs113233720	17	38008190				
**14**	**1**	rs112677036	17	38002152				
**15**	**2**	rs67600807	17	38001558				
**16**	**2**	rs9908694	17	37997771				
**17**	**2**	rs9900541	17	37996070				
**18**	**1**	rs111691913	17	37993238				
**19**	**2**	rs28449671	17	37991630				
**20**	**1**	rs111944912	17	37988476			**GH17J039817**	**37974070-37978821**
**21**	**2**	rs73304123	17	37987588				
**22**	**2**	rs112141468	17	37987464				
**23**	**1**	rs111734595	17	37987399				
**24**	**1**	rs113479772	17	37987042				
**25**	**1**	rs112797570	17	37983751				
**26**	**1**	rs112437508	17	37983512				
**27**	**2**	rs35130019	17	37983141				
**28**	**1**	rs111469562	17	37982696				
**29**	**2**	rs12942660	17	37982037				
**30**	**2**	rs8076347	17	37977540				
**31**	**2**	rs9908983	17	37976926				
**32**	**2**	rs9911069	17	37976601				
**33**	**2**	rs9901917	17	37976205	**5′ (I3)**	**37965773-37976506**		
**34**	**1**	rs112743130	17	37975855				
**35**	**2**	rs34053394	17	37975660				
**36**	**2**	rs58075375	17	37975592				
**37**	**2**	rs9902621	17	37973010				
**38**	**2**	rs9898031	17	37972647				
**39**	**1**	rs112412105	17	37971635				
**40**	**1**	rs113115305	17	37970686			**GH17J039812**	**37968642-37971311**
**41**	**1**	rs112238900	17	37968494				
**42**	**2**	rs67135646	17	37967871				
**43**	**2**	rs114777282	17	37967649				
**44**	**2**	rs4337325	17	37964435				
**45**	**2**	rs9901617	17	37964175				
**46**	**1**	rs113064843	17	37960421				
**47**	**2**	rs7211998	17	37959788				
**48**	**2**	rs36097841	17	37958112				
**49**	**2**	rs34988504	17	37957631	**3′ (E4-7)**	**37932293-37957717**	**GH17J039798**	**37954998-37957986**
**50**	**1**	rs16965347	17	37957566				
**51**	**2**	rs12937330	17	37957316				
**52**	**2**	rs34344462	17	37955193				
**53**	**2**	rs9899345	17	37954757				
**54**	**1**	rs113369293	17	37952654			**GH17J039790**	**37946728-37952847**
**55**	**1**	rs75148376	17	37952508				
**56**	**2**	rs73302152	17	37952350				
**57**	**2**	rs113159227	17	37952091				
**58**	**2**	rs56928975	17	37952031				
**59**	**2**	rs12938749	17	37951847				
**60**	**2**	rs35938199	17	37950812				
**61**	**2**	rs35105110	17	37950421				
**62**	**2**	rs35352075	17	37949790				
**63**	**1**	rs112771646	17	37945708				
**64**	**1**	rs112301322	17	37944518				
**65**	**2**	rs35088469	17	37944481				
**66**	**2**	rs34291217	17	37944410				
**67**	**2**	rs9911688	17	37943800				
**68**	**2**	rs9911669	17	37943766				
**69**	**1**	rs111862642	17	37942983				
**70**	**2**	rs34599546	17	37942971				
**71**	**1**	rs112345383	17	37942017				
**72**	**2**	rs1510475	17	37941379				
**73**	**2**	rs113812449	17	37940167				
**74**	**2**	rs9909365	17	37939958				
**75**	**2**	rs34016964	17	37938976			**GH17J039766**	**37922530-37939749**
**76**	**2**	rs67605703	17	37938496				
**77**	**2**	rs35506518	17	37938093				
**78**	**2**	rs13380871	17	37936248				
**79**	**2**	rs7224641	17	37934910				
**80**	**2**	rs12709364	17	37933822				
**81**	**1**	rs113370572	17	37933467				
**82**	**2**	rs9901483	17	37932773				
**83**	**2**	rs9894898	17	37932220				
**84**	**2**	rs9913596	17	37932062				
**85**	**2**	rs9652840	17	37929427				
**86**	**2**	rs71369788	17	37927144				
**87**	**2**	rs8072612	17	37927119				
**88**	**2**	rs9894370	17	37926003				
**89**	**2**	rs34758895	17	37925467				
**90**	**1**	rs112771360	17	37923770				
**91**	**1**	rs112876941	17	37922803				
**92**	**2**	rs2941509	17	37921193				
**93**	**2**	rs67571561	17	37920846				

**Table A6:** Allele-Specific Binding of Transcription Factors to Risk Alleles at *IKZF3* for which MAF_EUR_ > MAF_AFR_ but which are not included on the ImmunoChip.

Group I Risk Variants						*SNPs in IKZF3*-*ZPBP2* bi-Directional Promoter		
Risk SNP		Location	Interact. Fragment	ASTF	Alt-Ref Enrich.	Promoter SNP	Shared Promoter TF	Alt-Ref Enrich.
**A**	**rs193004755**	I1	*no*	-	−	-	-	−
**B**	**rs115164861**	I1	*no*	-	−	-	-	−
**C**	**rs142142756**	I1	*no*	Foxj1_1	−2.5	rs145735506	Foxj1_1	11.8
				Foxo_3	−2.3	rs184525456 rs138959946	Foxo_3	−12 −3
				p300_disc3	2.0	rs188089973 rs9907794 rs116467677 rs145275643 rs138461720 rs112745149 rs192412458	p300_disc5 p300_disc5 p300_disc9 p300_disc10 p300_disc5 p300_disc5 p300_disc1	1.9 −5.9 −1.7 11.9 −2.5 3.2 11.9
**D**	**rs145168309**	*I2*	*no*	AP-1_disc8	11.2	rs190729974 rs4795397 rs192412458 rs192412458 rs147224870 rs1453558 rs1453560 rs36111081 rs66565390	AP-1_disc1 AP-1_disc2 AP-1_disc3/7/9 AP-1_known2/3/4 AP-1_disc7 AP-1_disc2 AP-1_known1 AP-1_disc7 AP-1_disc7	12 −6.8 11.9/0.4/12 11.8/4.2/12 −10.9 11.9 −2.5 11.1 −11.1
				Irf_known7	9.0	rs9907564 rs188089973 rs75027016 rs138461720 rs112745149 rs184525456	Irf_known9 Irf_disc5/known9 Irf_known1/2 Irf_disc3 Irf_disc3/known9 Irf_known1/9	−1.1 11.9/−0.6 11.9/12 5.5 9.6/12 12/11.9
				Pax-5_disc4	4.7	-	-	−
				Pou2f2_disc1 Pou2f2_known10	4.7 3.1	rs202227901 rs191534721 rs9905881 rs193079571 rs140511615 rs4622539 rs184966935 rs145101657 rs145975450	Pou2f2_known4 Pou2f2_known4 Pou2f2_known2 Pou2f2_known2 Pou2f2_known8 Pou2f2_known8 Pou2f2_known2 Pou2f2_known10 Pou2f2_known2	−0.2 −0.6 2.6 4.3 −5.4 −5.2 1.9 4.9 1
				p300_disc5	2.9	rs188089973 rs9907794 rs116467677 rs145275643 rs138461720 rs112745149 rs192412458	p300_disc5 p300_disc5 p300_disc9 p300_disc10 p300_disc5 p300_disc5 p300_disc1	1.9 −5.9 −1.7 11.9 −2.5 3.2 11.9
**E**	**rs111907649**	*I3*	*no*	AP-1_disc7	−10.9	rs190729974 rs4795397 rs192412458 rs192412458 rs147224870 rs1453558 rs1453560 rs36111081 rs66565390	AP-1_disc1 AP-1_disc2 AP-1_disc3/7/9 AP-1_known2/3/4 AP-1_disc7 AP-1_disc2 AP-1_known1 AP-1_disc7 AP-1_disc7	12 −6.8 11.9/0.4/12 11.8/4.2/12 −10.9 11.9 −2.5 11.1 −11.1
				BHLHE40_disc2	−11.2	rs145275643 rs11557466	BHLHE40_known1 BHLHE40_known1	−0.2 1.3
**F**	**rs140386398**	*I3*	*no*	BDP1_disc3	−12	rs79042302	BDP1_disc1	−5.3
				GR_disc5	−12	rs199994111 rs183478341 rs192412458 rs190942850 rs192800564 rs11655198	GR_disc6 GR_disc1 GR_disc2 GR_known3/9 GR_disc6 GR_disc4	−0.3 6.6 11.8 −0.2/−0.3 −9.2 12
**G**	**rs149317842**	*I3*	*3′ E4-7*	Dlx2	−10.1	rs191534721	Dlx2	−1.9
				Dlx3	−9.4	rs191534721	Dlx2	−1.3
				Irx	−5.6	-	-	−
				Lhx3_1	−12	rs138350717	Lhx3_1	−1
				Pou3f2_2	−11	rs202227901 rs182045388 rs200781948 rs11078924	Pou3f2_2 Pou3f2_2 Pou3f2_2 Pou3f2_2	−12 −11 −12 −2.9
				SRF_known3	4.3	rs188089973 rs75027016	SRF_known3 SRF_known3	−1 1.3
				STAT_known3	4.9	rs202227901 rs191534721 rs4622539 rs145275643 rs79042302 rs79042302 rs112745149 rs192412458 rs181849193 rs185870642 rs145975450 rs74805134	STAT_disc5/known1 STAT_disc5 STAT_disc4 STAT_known13 STAT_disc1 STAT_known10/11/12/15/4/6/7 STAT_disc3 STAT_disc2 STAT_disc6 STAT_known14/15 STAT_known11 STAT_disc7	2.2/4.7 −11.8 12 5.2 −4 −11.9/1.2/−1/0.1/−3/−12/−0.9 12 12 11.9 11.9/11.9 −4.3 −11.7
				YY1_known6	3.7	rs188089973 rs147224870 rs28661251	YY1_known6 YY1_disc4 YY1_disc1/known2	−1.6 −3.1 −3.9/−0.6
**H**	**rs186234194**	*I7*	*3′ E4-7*	-		-	-	−
**I**	**rs145335424**	*I7*	*no*	AP-1_disc2	−12	rs190729974 rs4795397 rs192412458 rs192412458 rs147224870 rs1453558 rs1453560 rs36111081 rs66565390	AP-1_disc1 AP-1_disc2 AP-1_disc3/7/9 AP-1_known2/3/4 AP-1_disc7 AP-1_disc2 AP-1_known1 AP-1_disc7 AP-1_disc7	12 −6.8 11.9/0.4/12 11.8/4.2/12 −10.9 11.9 −2.5 11.1 −11.1
				Gfi1_3	−12	-	-	−
				NF-Y_disc1	−12	-	-	−
				NF-Y_known1	−5.2	-	-	−
				RFX5_disc2	−11.9	rs4795397	RFX5_disc2	−7.5
				TATA_disc6	−5.4	rs188089973 rs140511615 rs4622539 rs184966935 rs112745149 rs184525456 rs185009382 rs192678773	TATA_known4 TATA_disc9 TATA_disc9 TATA_known1 TATA_disc7 TATA_known1 TATA_disc7 TATA_disc7	0.7 −5.1 −3.2 −2.1 1.3 −0.6 −2.8 −7
**J**	**rs113730542**	*I7*	*no*	Fox	8.3	rs111678394	Fox	−1

**Table A7:** Risk Variants with Shared TF binding sites and Cell-type Specificity for DNAse I Hotspots.

SNP	DNAse HotSpot (ENCODE)	Interaction Region Hi-C	Shared TF between *IKZF3-ZPBP2* and 3′ (E4-7) Interaction Regions	Shared DNase HotSpot between *IKZF3-ZPBP2* and 3′ (E4-7) Interaction Regions	Source
rs111678394	y	*IKZF3*-*ZPBP2*	(Foxi1) Foxo_1, Pax-4_5	CD20, CD4, CD34+, **LCL**, **Th1**, **Th2**, **Treg**	Table 2
rs75148376	y	3′ (E4-7)	Ncx, Nkx6, Pou4f3, Dbx1, Hoxb4	**LCL**, **Th1**, **Th2**, **Treg**	Table 2
rs113370572	y	3′ (E4-7)	HDAC2	**LCL**, **Th1**, **Th2**, **Treg**	Table 2
rs113730542 *	y	<2kb from 3′ (E4-7)	Fox	CD4, **LCL**, **Th1**, **Th2**, **Treg**	Table A6
rs112876941	y	<10kb from 3′ (E4-7)	Foxa, Foxj1, Foxo, HNF1, TCF12	CD14+, **LCL**	Table 2

* rs113703542 is a risk allele from the EUR GWAS which was not typed on the ImmunoChip, so the variant was not included in Group 1 risk alleles, just in Table A5.
